# Structure-primed embedding on the transcription factor manifold enables transparent model architectures for gene regulatory network and latent activity inference

**DOI:** 10.1186/s13059-023-03134-1

**Published:** 2024-01-18

**Authors:** Andreas Tjärnberg, Maggie Beheler-Amass, Christopher A. Jackson, Lionel A. Christiaen, David Gresham, Richard Bonneau

**Affiliations:** 1 Center for Developmental Genetics, New York University, New York, NY 10003 USA; 2 Center For Genomics and Systems Biology, NYU, New York, NY 10008 USA; 3 Department of Biology, NYU, New York, NY 10008 USA; 4 Flatiron Institute, Center for Computational Biology, Simons Foundation, New York, NY 10010 USA; 5 Courant Institute of Mathematical Sciences, Computer Science Department, New York University, New York, NY 10003 USA; 6 Center For Data Science, NYU, New York, NY 10008 USA; 7 Prescient Design, a Genentech accelerator, New York, NY 10010 USA; 8 Mortimer B. Zuckerman Mind Brain Behavior Institute, Columbia University, New York, NY 10010 USA; 9 Sars International Centre for Marine Molecular Biology, University of Bergen, Bergen, Norway; 10 Department of Heart Disease, Haukeland University Hospital, Bergen, Norway; 11 Department of Neuro-Science, University of Wisconsin-Madison - Waisman Center, Madison, USA

## Abstract

**Background:**

Modeling of gene regulatory networks (GRNs) is limited due to a lack of direct measurements of genome-wide transcription factor activity (TFA) making it difficult to separate covariance and regulatory interactions. Inference of regulatory interactions and TFA requires aggregation of complementary evidence. Estimating TFA explicitly is problematic as it disconnects GRN inference and TFA estimation and is unable to account for, for example, contextual transcription factor-transcription factor interactions, and other higher order features. Deep-learning offers a potential solution, as it can model complex interactions and higher-order latent features, although does not provide interpretable models and latent features.

**Results:**

We propose a novel autoencoder-based framework, *StrUcture Primed Inference of Regulation using latent Factor ACTivity* (SupirFactor) for modeling, and a metric, explained relative variance (ERV), for interpretation of GRNs. We evaluate SupirFactor with ERV in a wide set of contexts. Compared to current state-of-the-art GRN inference methods, SupirFactor performs favorably. We evaluate latent feature activity as an estimate of TFA and biological function in S. cerevisiae as well as in peripheral blood mononuclear cells (PBMC).

**Conclusion:**

Here we present a framework for structure-primed inference and interpretation of GRNs, SupirFactor, demonstrating interpretability using ERV in multiple biological and experimental settings. SupirFactor enables TFA estimation and pathway analysis using latent factor activity, demonstrated here on two large-scale single-cell datasets, modeling *S. cerevisiae* and PBMC. We find that the SupirFactor model facilitates biological analysis acquiring novel functional and regulatory insight.

**Supplementary Information:**

The online version contains supplementary material available at 10.1186/s13059-023-03134-1.

## Background

Transcription factor (TF) regulation of mRNA transcription is a main mechanism through which cells control gene expression and respond to context-specific signals [1, 2]. The relationship between TFs and the genes they control forms an interconnected gene regulatory network (GRN), and interpreting this network is necessary to understand cell and organism heterogeneity, development and differentiation, tissue organization, and diseases [3–6]. GRNs are typically represented as causal graphs, which have regulatory TFs linked to target genes. Regulatory interactions in the GRN are difficult to collectively determine experimentally, so it is necessary to infer the structure of the GRN. This inference is complicated by regulatory relationships between TFs and genes that are context-dependent [7], as TFs may control gene expression differently in different cell types or under different conditions [8].

TFs themselves can be regulated transcriptionally (by changing their mRNA levels), translationally (by changing the amount of protein produced from mRNA), or post-translationally (by modifying the protein to alter localization or DNA binding ability). Transcription factor activity (TFA) can be thought of as a representation of how much a transcription factor influences the transcriptional rate of Pol II at its target genes, theoretically measured as the marginal rate of nascent transcripts produced per minute per target gene. To have activity, the TF protein must itself be expressed, and it must be able to localize to the nucleus and bind to its DNA target. Depending on the TF, it may require specific post-translational modifications, presence or absence of a small molecule, and presence or absence of co-activators or co-repressors. This TF activity is difficult to measure experimentally genome-wide.

Explicit inference of TFA as a latent model parameter is a core component of several GRN inference methods [9–13]. Generally, TFA is inferred from existing evidence of TF to target gene regulation, combined, using linear models, with the measured expression of the target genes. Although powerful, this framework lacks the flexibility to account for heterogeneity and contextual relations observed in biological systems, and the activity estimates have limited interpretability. Workarounds to contextualize regulatory relationships have been proposed [14, 15]; however, the inflexibility of the models and the lack of interpretability of latent factors remain an issue.

Using more complex models to better match known transcriptional regulatory biology places numerous demands on optimization and inference machineries and limits scale. However, the field of deep learning offers scalable learning and optimization techniques that could aid in GRN inference. Deep learning has been used to model expression and covariance networks [16], to build a sparse representation of gene clusters [17], to group genes into co-regulated modules [18, 19], to do supervised clustering of gene sets [20, 21] and for dimensionality reduction and denoising [22]. However, interpretable deep learning models for gene regulation, which provide biological insight into causal relationships in addition to prediction, have been difficult to construct and is an active area of research [23]. Techniques to interpret deep-learning latent features often focus on removing latent features to quantify their influence and importance [24]. Feature quantification can use change in mean squared error on removal of input features (COM) [25], backpropagation of the output layer into latent features (Grad-CAM) [26], or forward propagation of the latent layers into the output layer [19]. These methods often require a target value, e.g., as in classification tasks, or cannot be compared reliably between output features as non-normalized error rates depend on feature scaling. These techniques cannot be used for model selection. To make statements on the active GRN in a given context, we need to be able to determine predictive and non-predictive interactions. For unbounded metrics, this remains a problem as we need to construct a secondary heuristic on what constitute predictive and non-predictive bounds. Deep learning models generally remain over-parameterized, making biological interpretation difficult, and techniques must be applied after training to eliminate model parameters and enforce sparsity for GRN evaluation [27–35].

Knowledge priming embeds existing structural evidence into the model architecture by limiting connectivity between features based on epigenetic or regulatory evidence found in the literature. This informs the model of constraints on regulation which are complementary and not directly measured by the readout of gene expression in the training data. These constraints allow direct interpretation of the model, as priming with biological interactions allows biological interpretation of the resulting network graph [36]. Other structure evidence embedding methods use this prior data type as a constraint on covariance [37]. Both of these methods lack the ability to infer novel regulatory structure.

## Results

We present a model inspired by white-box machine learning approaches [38], that we call *StrUcture Primed Inference of Regulation using latent Factor ACTivity* (SupirFactor). This model incorporates knowledge priming by using prior, known regulatory evidence to constrain connectivity between an input gene expression layer and the first latent layer, which is explicitly defined to be TF-specific. This model ties the latent TF layer causally to *informative genes*, genes that have known upstream regulators given an independent set of evidence derived from literature or epigenetic data as described in “The SupirFactor regulation model” section, and allows the activation of latent features in this layer to be directly interpreted as transcription factor activity (TFA). This latent layer is then linked to gene expression in an output layer, which is interpreted as an explicitly inferred GRN.

We also adapt new metrics for model interpretation in this context; we define explained relative variance (ERV), a novel concept to interpret the structure of the inferred network graph for any architecture. Briefly, ERV is defined as the change in residual variance when a latent feature is removed from the model, and is used to rank and interpret graph weight importance within the model. Using ERV allows TF to gene interactions to be interpreted through additional latent layers placed between the TF latent layer and the output layer.

Benchmarking across multiple datasets we find that SupirFactor outperforms previous methods using similar frameworks for recovering GRNs. We find that our model uncovers biologically relevant TFA and predicts biological function of latent aggregates of TFs in deeper layers, suggesting our model is useful for predictive analyses beyond inferring GRNs. In particular we expect to predict activity of specific TFs and to aggregate TFs into regulatory pathways, which we demonstrate on an experimental *S. cerevisiae* data set and a mammalian large single cell PBMC dataset. GRN interpretability and context-specific network analysis is facilitated by using ERV, and we demonstrate its utility by applying trained models to context-specific and unseen data.

### The SupirFactor regulation model

The SupirFactor model learns a set of weights connecting genes to TFs in the prior. These genes functionally serve as reporters for the activity of connected TFs. As opposed to inferring TFA explicitly, as in network component analysis (NCA) (see the “Network component analysis (NCA)” section) where regulatory evidence is static in the prior, we set $\varvec{\phi } = g(\varvec{W}, \varvec{x})$, where $\varvec{W} \in \varvec{P}$ is the weighted influence of genes to TFs derived from prior evidence $\varvec{P}$, and $\varvec{x}$ is the expression of genes informing on $\varvec{\phi }$ which may be a subset of *informative genes* connected through $\varvec{W}$. This extends our model to $\varvec{x} = f(g(\varvec{W}, \varvec{x}), \varvec{\Theta })$, where $\varvec{\Theta }$ is the GRN.

**Fig. 1:**
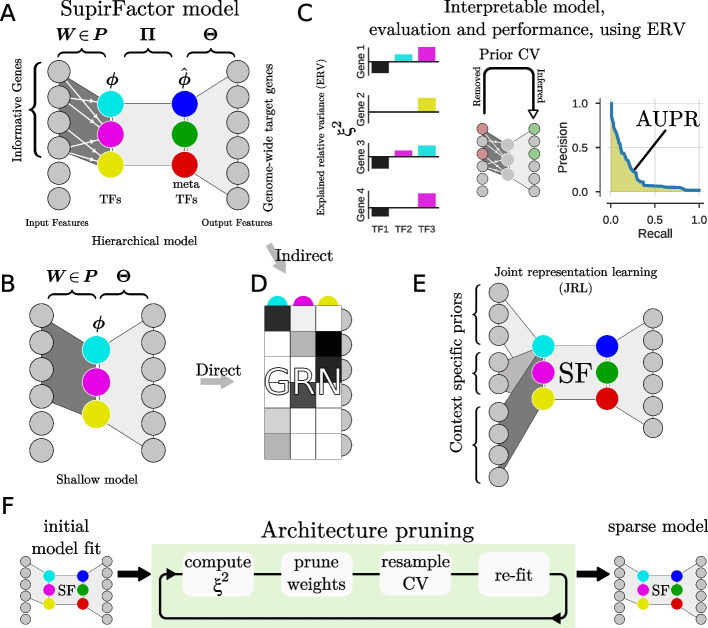
Outline of the SupirFactor framework. The SupirFactor model is constructed like an autoencoder where we embed gene expression data on the transcription factor manifold, exploring two architectures, the “hierarchical” **A**, and the shallow **B** architecture. The output of the first layer defines the latent features marked as TFs (Transcription Factors) and the activation $\varvec{\phi }$ is the transcription factor activity (TFA). The prior $\varvec{P}$ connect the evidence of TF to a set of informative downstream genes, with learnable weights $\varvec{W}$. For **A**, $\varvec{\Pi }$ connects the TFs to the latent features, here called the meta TFs (mTFs). $\varvec{\Theta }$ weights the mTF activity (mTFA) to predict genome wide gene expression profiles. In **B**, the TFs directly weights TF to gene influence in $\varvec{\Theta }$. **C**: To make the model completely interpretable and transparent we use explained relative variance (ERV) $\xi ^{2}$. ERV estimate importance of all latent factors influence on model output features. This is then used to evaluate the model and its performance. The GRN is cross validated, where genes to TF connections are held out in the input $\varvec{W}$ and predicted in the GRN which for the shallow model is $\varvec{\Theta }$ and for the hierarchical model is $\check{\varvec{\Theta }}$ the indirect effect from the TFs to output features. The measured recovery of these links gives insight on stability and biological relevance of the GRN where parameters are ranked by their predictability measured by $\xi ^{2}$. **D** Gene regulatory network extracted as indirect TF-gene interaction in hierarchical SupirFactor and direct TF-gene interactions in shallow SupirFactor. **E** Multi-task learning is implemented in SupirFactor through a joint representation learning (JRL) architecture where biological distinct contexts is independently weighted into a joint GRN representation. **F** Architecture pruning and sparsity procedure in SupirFactor is used to stabilize and eliminate over-parameterization by eliminating non-predicting model parameters facilitated be ERV

We actualize this model using a deep learning framework choosing *f*, and *g* and learn $\varvec{W}$, and $\varvec{\Theta }$ with $\varvec{\phi }$ as a latent feature produced by the weighted output from $\varvec{x}$ mapped through a learned $\varvec{W}$. Depending on the form of *f*, and including non-linearities, we can learn additional higher order interactions and regulatory pathways (see the “StrUcture Primed Inference of Regulation using latent Factor ACTivity (SupirFactor)” section). The complex version of the model that can capture other interactions in *f* we call “hierarchical” SupirFactor (Fig. 1A). A simple version of this framework uses a single bottleneck layer for our function *f* we refer to as “shallow” SupirFactor (Fig. 1B).

Constructing a prior matrix $\varvec{P}$ is a challenging but essential task for including informative evidence of regulation. This step is also a way to integrate data types that can shed light on TF-target relationships. This matrix can represent previously known interactions, and it can also encode higher probability interactions derived from chromosome accessibility or TF-chromatin interactions (experimentally measured by ATAC-seq and ChIP-seq) [39, 40]. A more dense $\varvec{P}$ is likely to include more false positives and will therefore result in a noisier propagation of TF variance. A sparser $\varvec{P}$ is likely to have many false negatives, limiting the variance that the model is able to explain, resulting in an model that may be less predictive.

A concern is that prior connectivity $\varvec{P}$ rarely includes reliable sign or weight estimates. Inferring signs for $\varvec{P}$ from the direction of change after perturbations is technically difficult, as it requires perturbing all TFs included in the model. Relying on expression correlation to infer signs will conflate both indirect and co-varying regulation. We expect that refitting weights $\varvec{W} \in \varvec{P}$ dependant on $\varvec{\Theta }$ will mitigate these problems.

### Selection of SupirFactor hyperparameters

We evaluate the SupirFactor framework (Fig. 1) for GRN inference from bulk and single-cell RNA expression data. First, to test our model setup, explore interpretability, and compare performance to other models, we benchmark using multiple data sets where a partial ground truth network is available (called the gold standard in this work) on the “shallow” SupirFactor. We have previously assembled a GRN inference data package that consists of two prokaryotic *Bacillus subtilis* bulk RNA expression data sets (B1 and B2), two *Saccharomyces cerevisiae* bulk RNA expression data sets (S1 and S2), and one *S. cerevisiae* single-cell RNA expression data set (scY) [41]. This data package also includes a *Bacillus subtilis* gold standard network [10], covering 154 TFs and 4218 informative genes also used as the prior evidence, and a *S. cerevisiae* gold standard network [42], covering 98 TFs and 993 target genes, both derived from literature databases, we also use a *S. cerevisiae* prior evidence matrix with 150 TFs and 5578 informative genes used in previous work [41]. Single-cell RNA expression data is preprocessed (see the “Single cell pre-processing” section) and both bulk and single-cell data is feature normalized (see the “Feature normalization” section) prior to model fitting. For the SupirFactor model, the number of latent features used $|\Phi |=$ number of TFs available in the prior evidence.

To be able to extract a GRN from our model that gives us latent feature to output connections, we need to be able to interpret model weights, features, and their relative importance. This is difficult in multi-layer architectures, and weights may not be scaled with biological importance. Instead, we devise a metric to quantify the direct effect a latent feature has on its targets in the output, explained relative variance (ERV) $\xi ^{2}$ (Fig. 1C), with appealing properties for GRN inference. This metric is based on feature removal [24] and is computed as the coefficient of partial determination (CPD) [43]. We disconnect each latent model feature and compute the consequent effect on the output variance MSE, scoring the separated model feature against the full model prediction without retraining (see the “Model selection and model parameter ranking” section).

Deep learning models have a number of hyperparameters that needs to be tuned for optimal model performance. Two critical hyperparameters for GRN inference are the *weight-decay*, typically called the L2 penalty, and dropout, stochastic perturbation of the data during training to attenuate noise and improve model generalization (see the “Model regularization” section). We test dependence on these hyperparameters by searching for L2 and dropout (on input and latent features) with the simplified shallow SupirFactor model. Each hyperparameter value is tested by splitting expression data 50–50 into training and validation set, using $80\%$ of the gold standard network as prior network information for the model and holding out $20\%$ for scoring. Negative controls consist of either shuffling the data or the prior network. Area under precision recall curve (AUPR) is used to score the network structure against the gold standard network and $R^2$ is used to quantify prediction accuracy. Each configuration is rerun repeatedly and average performance is reported (Additional file 1: Figs. S2-S9).

We observe that in some cases (for S2 and B1 datasets), when increasing L2 beyond a specific value, $R^2$ decreases while AUPR increases (Additional file 1: Figs. S3 & S4). We interpret this as overfitting to the prior network structure and increasing recovery of the gold standard in cases where the these two structures align, such as in scale-free networks with dense highly connected TFs. The model then emphasizes these TFs at the cost of prediction accuracy and inclusion of less connected TFs. This is undesirable, so we select hyperparameters that maximizes $R^{2}$ while maintaining a high AUPR. Negative controls perform as expected; shuffling the data eliminates the biological interpretability and predictive power of the resulting GRN, and shuffling the prior network eliminates only biological interpretability while still achieving good predictive power.

To determine an optimal L2, we look for where $R^2$ is maximized. We find that prediction accuracy is maximized in the span L2 $\in [10^{-6}, 10^{-4}]$ for all data sets, where we select an L2 of $10^{-4}$ for B1 and B2 and $10^{-6}$ for S1, S2 and scY for further comparisons (Additional file 1: Figs. S2C-S9C). For dropout, in general, we find that setting larger dropout on input and smaller dropout on latent features increases AUPR while maintaining a higher $R^{2}$.

### SupirFactor benchmarking demonstrates improved biological regulatory network recovery

**Fig. 2:**
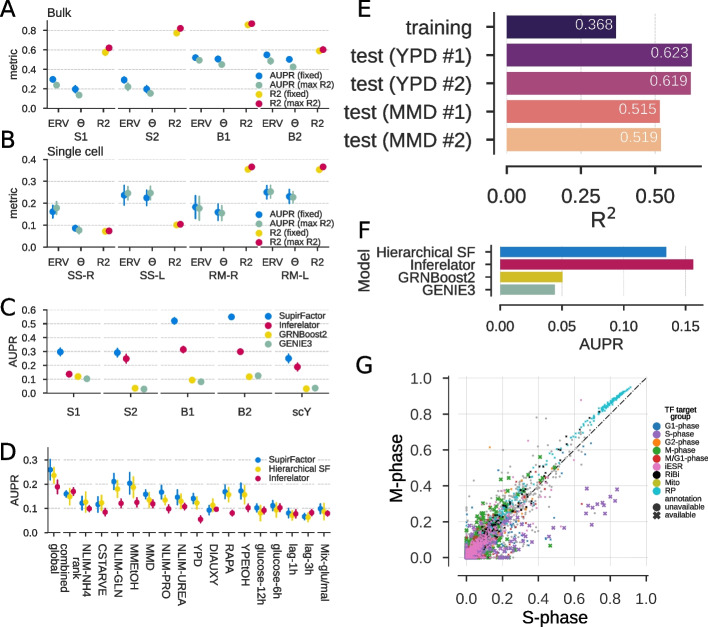
SupirFactor benchmark and hyperparameter evaluation. Performance is evaluated on gold-standard networks consisting only of edges held out of the prior network $\varvec{P}$, measuring recovery using AUPR. $R^{2}$ is computed on a validation set of $50\%$ of the data samples held out of the training data. Data sets are labeled for species, with *Bacillus subtilis* (B1 and B2), *S. cerevisiae* bulk RNA expression (S1 and S2), and *S. cerevisiae* single-cell RNA expression (scY). **A** Comparing network interpretation using model weights ($\Theta$) to interpretation using explained relative variance (ERV), measured using AUPR against edges held out of the prior network $\varvec{P}$. $R^{2}$ is calculated from the full network model. Model hyperparameters are set based on results in Additional file 1: Figs. S2-S9 as detailed in the “Model regularization” section. **B** Comparing normalization and activation functions for single-cell RNA expression data, as in (**A**). SS- is normalizing to mean of zero and unit variance, RM- is normalizing to maintain a minimum value of 0 (retaining sparsity), -L is linear activation, and -R is ReLU activation. **C** Benchmarking SupirFactor with optimal parameters selected from (**B**) against a comparable GRN inference method, the Inferelator, and two methods not using prior evidence; GRNBoost2 and GENIE3. **D** Comparing multi-context network performance between shallow SupirFactor, Hierarchical SupirFactor, and the multi-task Inferelator. GRNs are learned from single-cell (scY) data, with context/task groupings determined by growth condition. Global GRNs are learned from the data without separate groupings (using StARS-LASSO for the Inferelator [41]). Context networks are computed post-training in SupirFactor and split here on growth condition. **E** Evaluating model prediction $R^2$ on four novel test data sets, using a GRN trained by Hierarchical SupirFactor. **F** Recovery of independently collected regulatory evidence not in the prior. Comparing the full Hierarchical SF with the Inferelator, GRNBoost2, and GENIE3 on using the trained single cell Yeast (scY) models. **G** Comparing contextual network for GRNs defining cell cycle M-phase and S-phase (Table 1). Each point is an interaction from the two contextual networks, colored by the target gene functional annotation. *X* and *Y* axis are $\xi ^{2}$ of the S-phase and M-phase networks. GRN interactions targeting S-phase genes (purple) have higher ERV in the S-phase contextual network, and interactions targeting M-phase genes (green) have higher ERV in the M-phase contextual network

We summarize the performance of the shallow SupirFactor model using a linear activation function with several modeling choices (Fig. 2A). Using ERV as an estimator for biological relevance outperforms interpretation based on model weights alone, as determined by AUPR. Selecting the optimal dropout hyperparameters based on maximum $R^2$ for the selected L2 (max R2) improves model $R^{2}$ at a cost of decreasing network prediction performance (AUPR), when compared to setting a fixed dropout (fixed; input = 0.5 and latent = 0).

For single-cell RNA expression data (Fig. 2B), we extend the comparisons, comparing linear and rectified linear (ReLU) activation functions (see the “ReLU in hierarchical SupirFactor” section), and comparing standard normalization to a robust sparsity-retaining normalization (see the “Feature normalization” section). RobustMinScaler outperforms StandardScaler normalization, implying that preserving sparse data for model training is advantageous (Fig. 2B). To summarize these benchmarking results, ERV should be used to evaluate model parameters, and dropout hyperparameters can be fixed without loss of prediction accuracy and GRN recovery.

Finally, we compare shallow SupirFactor performance to that of the Inferelator (Fig. 2C), a method which takes comparable RNA expression and prior network inputs and learns a GRN. We note that other methods which take these inputs are not amenable to scoring on holdouts that we are employing for our benchmark [41], making comparison difficult. Adding prior evidence implies a causal chain defined by the prior, resulting in a better representation of regulation in the model. To test this assumption, we also benchmark against two state of the art methods using covariance of expression of a regulator to its targets, GRNBoost2 [37] and GENIE3 [34]. Using the configurations from our benchmarking, SupirFactor improves significantly over previous methods results on all of the data sets tested. Including prior evidence significantly improves network reconstruction.

### Hierarchical SupirFactor with non-linear activation facilitates interpretation of latent feature activation as biological activity in cell contexts

To account for more complex, multi-TF regulatory interactions, we extend our mode in multiple ways, introducing non-linearities and adding additional latent layers that represent interactions. Non-linear functional forms are necessary in the proposed hierarchical SupirFactor architecture to model interactions more complex than linear relationships. We add an extra latent layer with number of latent features $|\hat{\Phi }| = |\Phi |$ as shown in Fig. 1A. This is necessary as GRNs are context- and cell-type-dependent, and thus, a TF to gene regulatory interaction may, e.g., exist when the organism is in one state, but be inaccessible in another or change its regulatory role, e.g., activating or inhibiting, depending on its regulatory partners.

SupirFactor can distinguish contextual networks by embedding context-specific assigned data and computing ERV only within that data set. We explore learning context-dependent GRNs here; we evaluate this ability on the single-cell RNA expression data set, which has samples annotated by growth conditions (Fig. S1A). We compare hierarchical SupirFactor (Fig. 1A), shallow SupirFactor (Fig. 1B), and a comparable multi-task learning approach (AMuSR) in the Inferelator that also learns context-dependent networks. Both the shallow SupirFactor and hierarchical SupirFactor outperform the Inferelator (Fig. 2D). Shallow SupirFactor outperforms the hierarchical SupirFactor in some contexts, although the shallow model uses a linear activation function, and the hierarchical model uses a ReLU activation function. As this activation function constrains the latent features to be strictly positive, latent features are interpretable in the hierarchical model.

We use hierarchical SupirFactor to construct context-specific GRNs for cell cycle phases, by inferring cell cycle phase from transcriptional markers (Fig. S1B). Regulatory edges that are actively used in a context-specific GRN should explain more relative variance, compared to edges which are inactive. For each cell cycle, we used the gene annotation of cell cycle phase genes and compare ERV between phases for each relevant gene (Table 1). We compare both neighboring phases and phases skipping the immediate following phase. Computing a one-sided fisher exact test classifying genes in the GRN where they have the highest ERV, only using ERV that has $\xi ^{2} > 0.01$ in at least 1 of the conditions, we find that for all comparisons we have a strong enrichment of the phases relevant genes in terms of ERV in the corresponding phases.

**Table 1:**
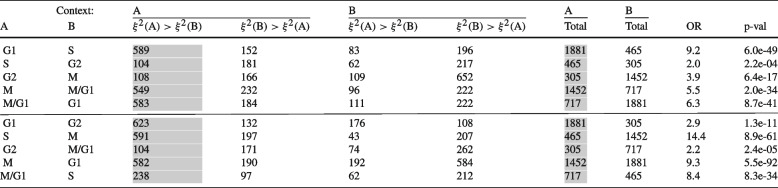
Pairwise comparisons between context-specific cell cycle phase networks from hierarchical SupirFactor. Network edges are classified into cell cycle phase context based on the existing annotation of the target gene. ERV of the network edges (where $\xi ^{2} > 0.01$ in one or more contexts) is then compared as a contingency table. Network edge counts in gray columns are edges where ERV is higher in the correct context-specific network, based on existing annotations. The total column counts network edges (where $\xi ^{2} > 0$ for that context) for the context-specific network and does not total the columns of the contingency table. Odds ratio (OR) and *p*-values are calculated by one-sided fisher exact test, using the contingency table

Hierarchical SupirFactor introduces a larger set of learnable weights and a potential over-parameterization of the model, and thus, the expanded model presents new model selection challenges in the context of sparse GRN inference. We test if an iterative method for removing model weights could be used without loss of model performance. ERV is used to rank model weights, and an $\xi ^{2} \le 0$ indicates a non-predictive model weight. Identifying these non-predictive links, pruning them, and then refitting the model attenuate over-parameterization (Fig. 1E). After 1 iteration of pruning, nonzero weights reduce to $\sim 57\% \in \varvec{\Theta }$, $\sim 52\% \in \hat{\varvec{\Theta }}$ and $\sim 80\% \in \varvec{\Pi}$ of the pre-pruning model trained on scY. It is critical to determine if models learned by hierarchical SupirFactor generalize. We evaluate the predictive ability of a hierarchical SupirFactor model trained on data set scY using prior knowledge $\varvec{P}$ without holdouts (Fig. 2B, RM-R). To do this, we generate four new experimental test single cell RNA expression data sets by collecting and sequencing cells grown in environmental conditions seen in the training data set (YPD and MMD). This model explains the variance of the training data well ($R^{2} = 0.37$), and also explains the variance of the YPD ($R^{2} = 0.62$) and MMD test data sets ($R^{2} = 0.52$) well (Fig. 2E).

Finally, we wanted to validate on regulatory edges not in the prior. We assembled an independent set of regulatory edges (see the “Yeast test GRN” section) and removed the overlap with the prior used for training. With this independent set of regulatory evidence, we evaluated each method’s model performance with AUPR. We found that the prior based method models outperform the other methods tested for the independent set (Fig. 2F).

We conclude that SupirFactor generalizes and predicts expression patterns of new data even when model weights have been removed.

**Fig. 3:**
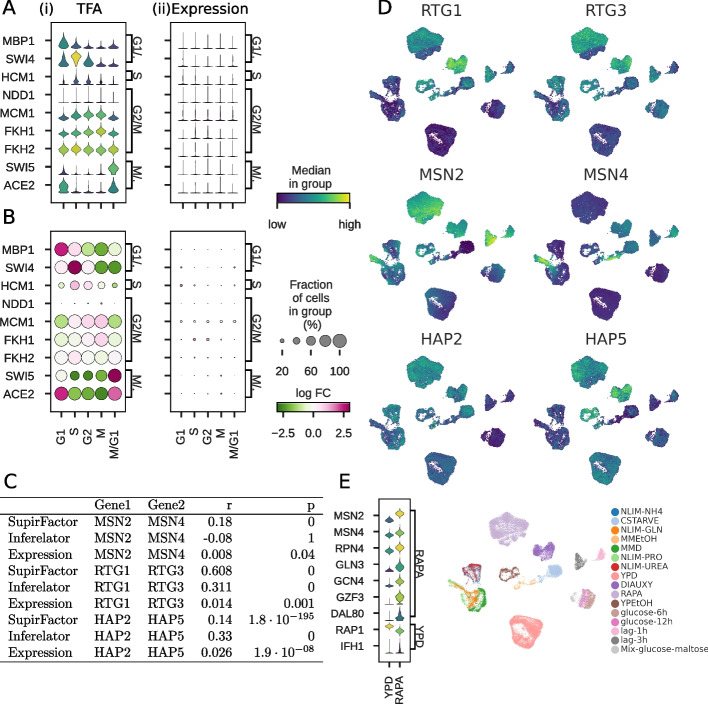
Transcription factor activity in single cell yeast. TFA estimated from hierarchical SupirFactor model. Violin plots are generated by scaling [0, 1] the underlying measurement. **A** Cell cycle TFs regulate gene expression in specific cell cycle phases, with the phase the TF regulates annotated on the right *y*-axis [45]. Cell cycle phase of each cell is inferred from transcriptome and annotated on the *x*-axis. Panel (i) plots TFA, and panel (ii) plots the RNA expression of the TF. **B** Same as **A**. Dot size represents the percentage of cells with a non-zero value. Color represents log fold-change (log FC) across the cell cycle phases. Panel (i) plots TFA, and panel (ii) plots the RNA expression of the TF. **C**: Interacting TF pair Pearson correlation for SupirFactor TFA, Inferelator TFA, and TF expression. **D** Comparing TFA between rapamyacin (RAPA) treated and untreated (YPD) cells TFs known to be activated by treatment [46] or known to be more active in untreated cells are annotated on the right *y*-axis. **E** UMAP projection of the scY dataset showing TFA estimate of co-regulator TFs and growth conditions

The SupirFactor model explicitly fits an intermediate layer which can be directly interpreted as latent Transcription Factor Activity (TFA) for each TF, when this layer uses a ReLU activation function [44]. Using hierarchical SupirFactor, we calculate latent TFA for all TFs. When examining the role of cell cycle TFs, the advantages of TFA are apparent. The TFA for cell cycle TFs is maximal in phases the TFs are expected to regulate (Fig. 3A), based on known TF roles from literature [45]. Almost all cells have non-zero TFA for cell cycle TFs in at least one phase of the cell cycle, but TF expression is highly sparse, complicating causal linkage to targets based on TF expression (Fig. 3B). We note that for these cell cycle TFs, the expression of the TF often peaks in the phase before the TFA of the TF.

We also wanted to test the role of functionally related TFs. Transcription factors often interact with each other to define regulatory states, as part of multi-subunit complexes, or by competing for the same DNA binding regions. These interactions should be reflected in how TF activity correlates, even if these interactions are not explicitly embedded in the model. We select several typical examples of TF pairs with known interactions and compare inferred TFA between hierarchical SupirFactor and the Inferelator (Fig. 3C). MSN2 and MSN4 are partially redundant stress response TFs that bind to the same DNA motif, and we expect their activity to be partially correlated. Hierarchical SupirFactor TFA is weakly correlated ($r=0.18$), unlike expression of MSN2 and MSN4, which are uncorrelated ($r=0.01$). RTG1 and RTG3 are obligated to form a physical dimer for functionality, and we expect their activity to be strongly correlated. Hierarchical SupirFactor TFA is strongly correlated ($r=0.61$), unlike expression of RTG1 and RTG3, which are uncorrelated ($r=0.01$). Finally, HAP2 and HAP5 are part of the multisubunit heme-activated TF complex, and we expect their activity to be strongly correlated. In this case, hierarchical SupirFactor is less successful at correlating TFA ($r=0.14$) than the Inferelator ($r=0.33$); expression is again uncorrelated ($r=0.03$). Overlaying TFA onto a reduced-dimensionality plot allows for the comparison between TF activities and the experimental conditions which cause them to be correlated or uncorrelated (Fig. 3D).

Finally, we compare the TFA between perturbed, rapamycin-treated cells and untreated control cells (Fig. 3E). Rapamycin is expected to inhibit TOR pathway signaling, altering stress response and nutrient response TF activities [45]. By comparing the TFA between perturbed and control cells, hierarchical SupirFactor is able to reconstruct which TFs are activated and deactivated by this perturbation.

### Hierarchical SupirFactor combines TFs into pathways

In hierarchical SupirFactor, we introduce an additional latent layer, which we interpret as meta transcription factors (mTFs) that aggregate TFs into multi-regulator pathways. As this mTF layer is directly connected to the output gene expression, we expect that the mTF layer activity (mTFA) can be interpreted as the activity of a regulatory pathway.

**Fig. 4:**
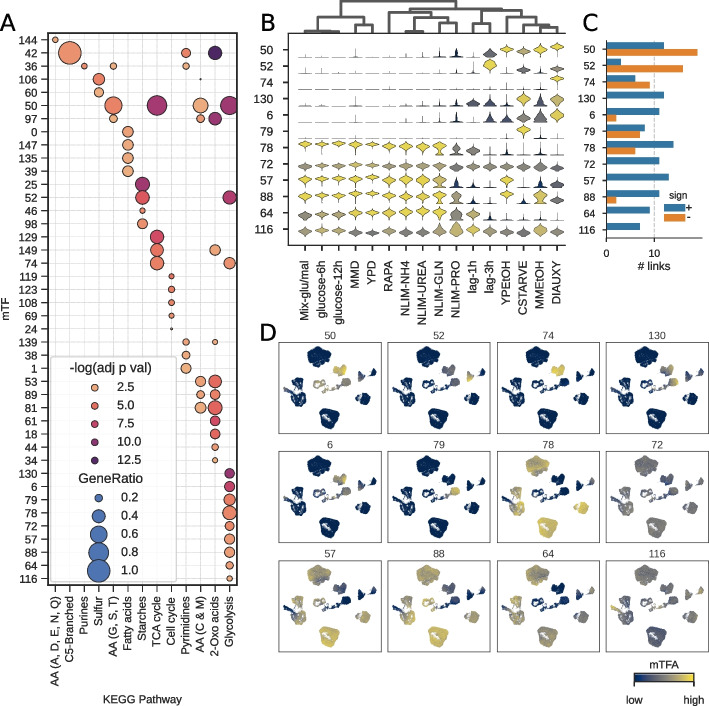
Meta transcription factor (mTF) functional enrichment analysis in single cell yeast. mTFA estimated from hierarchical SupirFactor model trained in Fig. 3. mTFs are nodes in the SupirFactor model $\check{\Phi }$ latent layer and are numbered from 1 to 148. **A** Pathway enrichment of mTFs to selected core metabolic KEGG pathway annotations on target genes. **B** mTF activity for cells in each growth condition (mTF activity scaled [0, 1] for comparison). **C** Positive (activating) and negative (repressing) weights from mTFs to target genes within the Glycolysis KEGG-pathway for each mTF. **D** mTF activity for cells overlaid on a low-dimensional UMAP projection. Cell metadata plotted in Additional file 1: Fig. S1

To test this hypothesis, we explore the hierarchical SupirFactor model trained on the single-cell yeast data (scY). mTF functions are determined by enrichment for regulation of genes that are annotated with Kyoto Encyclopedia of Genes and Genomes (KEGG) pathways [47]. As this data was collected from cells growing in different carbon and nitrogen sources, we focus on enrichment of a specific subset of metabolic pathways. Forty-two of 148 mTFs are enriched for target genes (defined as mTF to gene connections with $\xi ^{2} > 0.1$) to these metabolic KEGG pathways (Fig. 4A).

We focus on the 12 mTFs which are enriched for glycolysis target genes, a pathway which is a core part of the central carbon metabolism. Many of the growth conditions in the training single-cell yeast gene expression data set use glucose as a primary carbon source (Additional file 1: Fig. S1E), and we see that these conditions have similar mTF activation (for mTFs; 78, 72, 57, 88, 64, 116) within the glucose conditions (Fig. 4B). The remaining growth conditions use different carbon sources, requiring different regulation of the central carbon metabolism. Six of the glycolysis mTFs are activated in these non-glucose carbon sources, but have considerably more repressive (negative weighted) links to target genes (Fig. 4C), suggesting that they are mainly downregulating glycolytic genes. We can further overlay mTF activity onto a low-dimensionality projection in order to identify mTFs which are linked to carbon source with little heterogeneity (e.g., 74 and 79) and which have heterogeneity within growth conditions (e.g., 57 and 78) (Fig. 4D). We observe that mTFs aggregate biologically functional groups in their targets and can be evaluated quantitatively as activities of these pathways.

### SupirFactor model regulation of mammalian PBMCs with multimodal single cell sequencing data

**Fig. 5:**
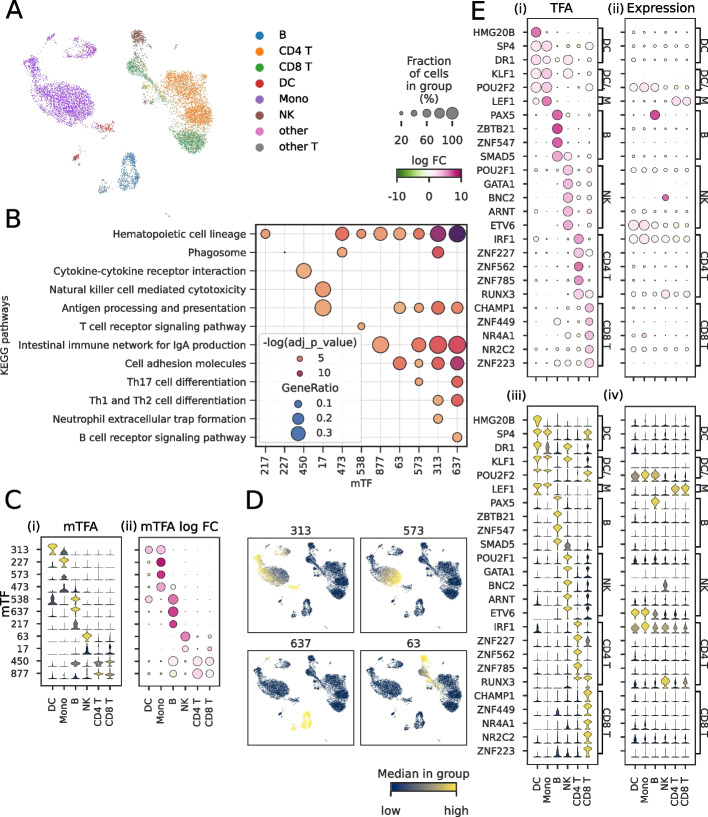
Single cell PBMC TFA and mTF functional enrichment analysis. TFA and mTFA are estimated from a JRL SupirFactor model. mTFs are associated with latent features $\hat{\phi }$ and are numbered 0-881. **A** UMAP projection of the single cell PBMC dataset, labeled with cell type annotations. **B** Selected enriched terms and associated mTFs for cell-type specific KEGG pathway based on mTF target genes, $\xi ^{2} > 0.01$ (for mTF 227, phagosome pathway, adjusted *p *= 0.0081, gene ratio = 0.0309). **C** Activity of functionally enriched mTFs, over each cell-type. **D** UMAP projection of PBMCs colored by mTF activation. **E** Significant TF activation for specific cell-type populations and corresponding gene expression (scaled [0, 1] for comparison). Dot plots of (i) TFA and (ii) TF mRNA expression. Violin plots of (iii) TFA and (iv) TF mRNA expression

We evaluate the use of SupirFactor to model complex biological systems by applying the method to model Peripheral Blood Mononuclear Cell (PBMC) gene regulation, using a paired multi-omic single cell ATAC-seq and RNA-seq dataset [48] (Fig. 5A). These two data types are integrated by using ATAC-seq chromatin accessibility as a cell-specific mask (Eq. 16).

PBMCs are a heterogeneous pool of multiple cell types, and each cell type may have subsets of cells in different states. We annotate these cells as dendritic cells (DC), monocytes (Mono), natural killer cells (NK), B-cells (B), CD4^+^ T-cells (CD4 T), and CD8^+^ T-cells (CD8 T). To account for this heterogeneity, we build a context aware joint representation learning (JRL) SupirFactor model (see the “Joint representation learning for context-specific constraints” section). This allows the model to weight regulatory evidence based on context and aggregate into a joint latent feature space to build a joint SupirFactor GRN model. We define cell contexts for JRL by clustering cells using Leiden clustering [49], with a resolution $=0.2$, generating 7 clusters (Additional file 1: Fig. S11A-B). The PBMC SupirFactor model was trained using JRL on ATAC masked data (epochs = 400) and subset to the most explanatory model weights once (epochs = 100), resulting in a model that is scored on held-out cells ($R^{2} = 0.35$). The modeled PBMC regulatory network predicts 818 active TFs regulating 13,698 genes, connected through 492 mTFs in a latent mTF layer ($\xi ^{2} \ge 0$) (Additional file 1: Fig. S11C).

Eleven of these mTFs are linked to target genes which are functionally enriched for immune cell specific KEGG pathways (Fig. 5B), and we interpret these mTFs as regulatory pathways. As an example, mTF-637 is active in B cells but largely inactive in other PBMCs (Fig. 5B-D); the target genes of this mTF are functionally enriched for B-cell receptor signaling (Fig. 5B). mTF 313 is activated in dendritic cells (DCs) and regulates genes functionally enriched for phagocytic activity and for neutrophil extracellular trap formation, which activate DCs and allow them to mature [50]. mTF 227 is also linked to phagocytosis, although is principally active in monocytes. mTF 17 is active in NK cells with functional enrichment for natural killer cell-mediated cytotoxicity genes. Overall, this demonstrates the utility of SupirFactor mTFs as a tool for identifying cell type-specific regulation.

We can further examine TFs that have inferred cell-type-specific activity (Fig. 5E). SupirFactor distinguishes activation between myeloid (monocytes and DCs) and lymphocytic (B, NK, and T cells) lineages (Fig. 5E). The framework also recovers known cell-type-specific regulators along the myeloid lineage, monocytes and DCs, (KLF1) [51], B cells (PAX5, SMAD5) [52, 53], NK cells (ARNT) [54], CD4 T cells (IRF1, RUNX3) [55, 56], and CD8 T cells (NR4A1) [57]. We show that SupirFactor infers cell-type-specific differential mTF activation and TF activation among distinct cell types that correspond with known biological processes and protein activity for multiple key cell types. The analysis of SupirFactor performance on the PBMC dataset demonstrates that SupirFactor can learn biologically relevant interactions in complex organisms and datasets.

We apply the trained PBMC model to single-cell PBMCs collected from healthy and from COVID-19-infected individuals [58] (Additional file 1: Fig. S13). This trained model computes TFA for each cell, allowing determination of differentially active TFs for each cell type (Additional file 1: Fig. S13A). Immune cells from COVID-infected patients show an upregulation of core inflammation TF activities (e.g., the Activator protein-1 TF components FosB and JUN), and changes to differentiation and proliferation TF activities (e.g., RUNX3 and IRF5). We further identify several TFs which are not, in healthy cells, specific to any immune cell type, but are predicted to have cell type-specific changes to TF activity during infection (e.g., HMBOX1, ZNF24, ZNF691). Based on this analysis, we conclude that exploring condition-specific data with SupirFactor models can uncover upstream causal regulators that are both cell-type and condition specific.

## Discussion

SupirFactor has been carefully benchmarked using both bulk RNA expression data sets and single cell RNA expression data sets. We rely on model organisms *Bacillus subtilis* and *S. cerevisiae* for benchmarking, as these organisms are well-characterized and have a partial experimentally validated ground truth network available, which we use for scoring recovery of GRN structure. This model organism benchmarking is important, as mouse and human data sets used for GRN inference benchmarking often lack reliable ground truth networks for scoring and are restricted to using predictive metrics which have limited value. This benchmarking shows that the SupirFactor framework is versatile and has improved GRN inference over a comparable framework that relies on statistical learning, measured by recovery of network edges which are held out of the modeling. The SupirFactor GRN models are also predictive, and we expect that future work will tune the model to optimize network recovery using AUPR, or other model selection appropriate metrics, of held out gold standard network edges, by maximizing *R*^2^ for predictive power.

Additionally, we provide a novel metric for evaluating deep neural networks (DNN) architectures, ERV, specifically designed for the needs of GRN inference. By using ERV to evaluate linkages from regulatory TFs, through a latent meta-TF layer, to target genes, we are able to use the meta-TF layer as a powerful pathway analysis tool.

We demonstrate the validity of ERV by comparing it directly to GRN inference using model weights alone and find that it improves GRN inference link interpretation. ERV also allows for post-training analysis of any gene expression data to determine which parts of the network is specific to that context. We show this by extracting context-specific networks from the *S. cerevisiae* single cell data set, which contains observations from fifteen different growth conditions. The recovery of these post-training context-specific networks is an improvement over previous work, which requires that the context is embedded into the model pre-training. SupirFactor is therefore a valuable tool to identify context specific and contextual regulatory interactions.

The driving features of GRNs can be condensed into TFs. Another core concept explored in this work is that of latent feature inference and interpretation, i.e., TFA. In the model organisms *S. cerevisiae*, we demonstrate that the model latent feature activity, the TFA of a TF, is distinct from the expression of that same TF. We do this by studying the cell-cycle where we see a clear delay of relevant TFA compared to expression. Demonstrating that using TF expression as the independent feature to model a GRN will not capture the regulatory structure of GRNs, a drawback in many works related to GRN inference.

To demonstrate that SupirFactor scales to the complexity of mammalian systems, we evaluate a model learned from a PBMC multi-ome single cell dataset and characterize the pattern of TFA and functional enrichment in different contexts. The model makes use of context-specific prior evidence to further restrict TF variance. And we find that we can extract functional enrichment based on annotated cell types reliably.

Reuse of computational models can be valuable as a tool to understand and conceptualize new experimental data evident by recent reuse of single cell sequencing atlases in the field of genomics [48, 59]. Unfortunately, the reuse of GRNs themselves is rare, and for most studies, gene regulatory networks are inferred entirely based on new data. We consider this to be a general limitation in current-generation GRN inference models, which do not have mechanisms to embed new data into an existing GRN. SupirFactor tackles this issue by using a DNN architecture together with the transparency framework (ERV). We demonstrate this reuse by embedding new data and by contextually analyzing sub-networks and condition specific latent activation after the model has been trained, gaining insight not explicitly provided to the model before training.

## Conclusion

In this study, we describe *StrUcture Primed Inference of Regulation using latent Factor ACTivity* (SupirFactor), a model within the class of knowledge primed deep learning models. SupirFactor explicitly treats transcription factor activity (TFA) as an interpretable latent state which drives gene transcription. This model uses a single objective function where the influence of the prior regulatory structure is optimized together with the gene regulatory network (GRN). SupirFactor combines the power of DNN optimization with prior structure constraints for inferring GRNs and explicit estimation of TFA. These TFA estimates are bounded by a ReLU activation function and are directly quantifiable and interpretable on a per-observation basis.

Additionally, in this work, we devise the explained relative variance (ERV) metric. ERV estimates the importance of each latent feature to each output feature, both directly and indirectly depending on the model layer in question. This metric has appealing properties for GRN inference, e.g., ERV is bounded and facilitates ranking regulatory relationships and discarding non-predictive model weights when interpreting the model. As biological GRNs are sparse model selection must be part of the evaluation of any GRN inference algorithm and ERV facilitates this critical component. This model evaluation metric we propose is also useful for evaluating the contribution of intermediate DNN layers which are not explicitly defined as TFs and facilitate functional annotation and latent feature interpretation.

Developing explainable deep learning models for GRN inference is a critical requirement for improving models of gene expression and regulation [23]. The goal of this work was to build a formalized GRN inference model with explicit optimization and objective functions, from which latent states can be directly interpreted. The resulting formalism, SupirFactor, is a powerful GRN inference tool with additional pathway analysis and protein activity functionality, that can be applied to both bulk and single cell data. SupirFactor can harmonize regulatory evidence, epigenetic data and expression readout in a regulatory and functionally meaningful way. While challenges still exist, like model stability and model selection, tightly connected to the nature of non-linear machine learning algorithms, advances in single cell multi-omics and epigenetic sequencing are steadfast and will further narrow the specificity in model constraints with its inclusions. With additional work related to architecture, algorithm development, and prior evidence construction, the framework can be further extended and prove even more useful.

## Methods

### *StrUcture Primed Inference of Regulation using latent Factor ACTivity* (SupirFactor)

We define two SupirFactor models. The shallow model, used mainly for testing, which consists of a single layer that represents individual TFs and their activity. The hierarchical model, which consists of two layers, the first representing individual TFs and their activity, and the second representing TFs aggregated into pathways, termed meta TFs (mTFs). The hierarchical model is the main model used in this work.

#### Shallow SupirFactor

Starting from our model framework (see the “The SupirFactor regulation model” section), gene expression is a function of TFA and is used as the independent feature to weight influence on gene expression from TFs. We can formulate the problem as

1 $$\begin{aligned} \hat{\varvec{x}} = h(\varvec{x}) = (g \circ f)(\varvec{x}) \end{aligned}$$

where $\varvec{x} \in \mathbb {R}^{n}$ is the gene expression of one observation with *n* genes, with *g*, *f* as functions of the form

2 $$\begin{aligned} \varvec{\phi } = f(\varvec{x}) = \sigma \left( \varvec{W} \varvec{x} \right) \end{aligned}$$

and

3 $$\begin{aligned} \hat{\varvec{x}} = g(\varvec{\phi }) = \sigma \left( \varvec{\Theta } \varvec{\phi } \right) \end{aligned}$$

with the activation function $\sigma$, aggregating the linear combination of inputs for each latent feature. The linear combination of inputs without activation is similar to the NCA framework described in “Network component analysis (NCA)” section, with the distinction that this formulation weighs $\varvec{W}$ instead of fitting $\varvec{\phi }$ with a static $\varvec{P}$.

$\varvec{\Theta }$ and $\varvec{W}$ are the weighted connections of input features to output features in a single layer, with equation (3) corresponding to the shallow SupirFactor (Fig. 1C). $\varvec{\phi }$ is the inferred latent activity interpreted as TFA of expression mapped through *f*. We set $\varvec{W} \in \varvec{P}$, such that the sparsity structure of $\varvec{W}$ is identical to $\varvec{P}$. This ensures that (I) $\varvec{\phi } \in \mathbb {R}^{K}$, with *K* TFs, constraining the informative genes influences to those in the prior $\varvec{P}$, and (II) the causal flow from regulator to target defined by the prior is enforced. Causality does not imply direct binding but rather in this case the variance of each TF being constrained to the variance of its targets as opposed to the covariance of the TF expression.

#### Hierarchical SupirFactor

In hierarchical SupirFactor (Fig. 1A), we add an additional mTF layer between TFs and output gene expression. This allows higher order interactions between TFs (representing biological concepts like redundancy, competition, and physical complexing), and other conditional non-linear dependencies to be modelled. This extends the formulation of SupirFactor so that it can generate TF interaction hypotheses and be used as a tool for pathway analysis.

4 $$\begin{aligned} \hat{\varvec{x}} = h(\varvec{x}) = (g \circ s \circ f)(\varvec{x}) \end{aligned}$$

where

5 $$\begin{aligned} \hat{\varvec{\phi }} = s(\varvec{\phi }) = \sigma \left( \varvec{\Pi } \varvec{\phi } \right) \end{aligned}$$

and

6 $$\begin{aligned} \hat{\varvec{x}} = g(\hat{\varvec{\phi }}) = \sigma \left( \varvec{\Theta } \hat{\varvec{\phi }} \right) \end{aligned}$$

$\varvec{\Pi }$ is the weight matrix of TF-TF interactions that maps individual TF activity to the mTF layer.

#### Joint representation learning for context-specific constraints

Joint representation learning is a transfer learning method where context-specific evidence is aggregated into a common model structure (see [44], chap. 15) (Fig. 1E). This is implemented in SupirFactor by adding a biological context-specific constraint on the prior evidence. We define $\varvec{P}_{C}$ as prior evidence for *C* where *C* is a biological context, like a cell type, growth condition, or temporal group. Weights $\varvec{W}_{C} \in \varvec{P}_{C}$ are also context-specific and are mapped jointly through $\varvec{\Pi }$ and $\varvec{\Theta }$ that are common to all contexts. Experimental data is labeled with the appropriate context and data for each context is submitted batch-wise to the model for training. Context weights $\varvec{W}_{C}$ are individually trained and may vary between contexts if $\varvec{P}_{C}$ is the same. Equation (4) then takes the form;

7 $$\begin{aligned} \hat{\varvec{x}}_{C} = (g \circ s \circ f_{C})(\varvec{x}_{C}) \end{aligned}$$

where $f_{C}$ is the context specific structure ($\varvec{P}_{C}$) primed encoder.

#### Fitting model

To train the SupirFactor model, $\varvec{W}$, $\varvec{\Pi }$, and $\varvec{\Theta }$ are fit to minimize mean squared error between $\hat{\varvec{x}}$ and $\varvec{x}$.

8 $$\begin{aligned} \text {MSE} = \frac{1}{N} \sum _{i} \left\| {\varvec{x}_{i} - h(\varvec{x}_{i})}\right\| ^{2} \end{aligned}$$

This is implemented as minimization by batch stochastic gradient descent with the Adam solver [60] and pytorch [61].

Nonzero encoder weights are initially mirrored and made non-negative in both the decoder $\varvec{\Theta }$ and encoder $\varvec{W}$, although all elements in $\varvec{\Theta }$ are free to be fit by the solver. The prior assumption is that TFA is positively correlated with gene expression during model initialization if no other evidence is available.

### Model regularization

#### Parameter penalties

Model weights are penalized, regularizing models to mitigate overfitting and to balance bias and variance [62]. We use a *weight decay* factor, corresponding to a ridge penalty (see [63], Section 6.7.6). The objective function to be minimized is then

9 $$\begin{aligned} \text {MSE} + \zeta \left\| {\varvec{W}}\right\| _{2} + \zeta \left\| {\varvec{\Theta }}\right\| _{2} + \zeta \left\| {\varvec{\Pi }}\right\| _{2} \end{aligned}$$

where $\zeta$ is the ridge penalty applied. The $\zeta$ parameter is set by cross validation, splitting the data into equal training and validation sets and evaluating model performance where $\zeta \in \{0, 10^{-10}, 10^{-9}, \ldots , 10^{-1}\}$ [31, 64].

#### Dropout

Dropout is an additional regularization method [65] where a fraction of nodes are removed from each sample during training, mitigating the risk that noise in the data will trap the model in a local minimum. Dropout can be applied to input or to latent layers. In short, training data is randomly batched into groups, and each batch is then used to train a network where a fraction, *p* of data points are removed randomly from each sample in each batch before feeding it through selected layer(s). This is implemented in SupirFactor through the Dropout module in pytorch. Droput is tested by cross validation as above on both the input and the latent TF layer, searching $p \in \{0, 0.01, 0.05, 0.1, 0.2, 0.3, 0.4, 0.5\}$.

### Explained relative variance (ERV)

#### Model selection and model parameter ranking

GRNs are sparse and most genes have a limited number of directly regulating TFs. The importance of model parameters is quantified, and relatively unimportant parameters are shrunk to zero. Two different ways of ranking inferred interactions are evaluated in this work: (I), ranking the magnitude of model weights $|\theta _{i, k}|$ (MODEL), and (II) ranking interactions by their explained relative error (ERV). ERV perturbs latent features and quantifies the consequence of that perturbation [35]. The goal is not to eliminate redundancy, but rather to eliminate over-parameterization and constrain the parameter space of SupirFactor.

ERV is calculated as coefficient of partial determination [43] so the bound on the error contributing to predict gene expression can be evaluated $\xi ^{2} \in ~]-\infty , 1]$ where a predictive link has an $\xi ^{2} \in ~]0, 1]$. The GRN model is trained once, as re-training for each perturbed latent feature is computationally intractable. ERV is determined from the ratio of the full model MSE to the perturbed model MSE.

10 $$\begin{aligned} \xi ^{2}_{i, k} = 1 - \frac{\text {MSE}_{i}{(\bar{h}(\varvec{x})_{\varvec{\phi }})}}{\text {MSE}_{i}{(\bar{h}(\varvec{x})_{\varvec{\phi }_{\textrm{K} \ne k}})}} \end{aligned}$$

$\bar{h}$ is the trained model, $\text {MSE}_{i}{(\bar{h}(\varvec{x})_{\varvec{\phi }})}$ is the MSE of the full, unperturbed model for gene *i*, and $\text {MSE}_{i}{(\bar{h}(\varvec{x})_{\varvec{\phi }_{\textrm{K} \ne k}})}$ is the MSE of the perturbed model for gene *i* where the activation of the latent layer for regulator *k* is set to 0. Model parameters for each gene *i* are ranked by the value of $\xi ^{2}_{i, k}$ for all *k*.

#### Ranking model parameters for models with multiple layers

To be able to interpret any parameter in the model we use the ERV concept (see the “Model selection and model parameter ranking” section). To determine the importance in any hidden layer *L* not directly connected to the output, we compute two $\xi ^{2}$ matrices. $\xi ^{2}_{L}$ and $\xi ^{2}_{L+1}$. Where *L* indicates the layer with corresponding latent feature input to the layer in question and $L+1$ the next layer with corresponding latent feature input. For layer weights $\varvec{\Pi }$ we compute each element $\pi _{m, k}$ with *m* as output feature and *k* as input feature by first computing the vectors $\xi ^{2}_{:, k, L}$ and $\xi ^{2}_{:, m, L+1}$. That is, the $\xi ^{2}$ of each element of $\varvec{\Theta }_{:, m}$ and the $\xi ^{2}$ of each element of the indirect contribution of latent feature *k* in layer *L* to the output genes $\xi ^{2}_{:, k, L}$. To eliminate weights in $\varvec{\Pi }$ we threshold $\varvec{\xi ^{2}}_{:, k, L+1} > \epsilon _{1}$ and $\varvec{\xi ^{2}}_{:, m, L}\ > \epsilon _{2}$ and compute the ERV:

11 $$\begin{aligned} \xi ^{2}_{m, k, \varvec{\Pi }} \equiv 1 - \frac{\sum _{i \in k \cap m}\text {MSE}_{i}{(\bar{h}(\varvec{x})_{\varvec{\phi }})}}{\sum _{i \in k \cap m} \text {MSE}_{i, k, L}{(\bar{h}(\varvec{x})_{\varvec{\phi }_{\textrm{K} \ne k}})}} \end{aligned}$$

where $i \in k \cap m$ is the intersection set of predictive TF to gene interactions for latent feature *k* from layer *L*, and *m* for layer $L + 1$. If $k \cap m = \emptyset$ then $\xi ^{2}_{m, k, \varvec{\Pi }} \equiv 0$.

The classical GRN realization is interpreted as the indirect connections from $\xi ^{2}_{i, k, L}$, connecting the latent input features in layer *L*, *k* to the output target genes *i*. With *L* in this case representing the TFA activation layer.

#### Stable architecture and non-predictive weight elimination

To select an interpretable model, we want to reduce the model size in terms of individual weights to arrive at a model with parameters that are predictive and stable. We define predictive as when an individual parameter that can be determined to connect an upstream regulator to its downstream target, has $\xi ^{2} > \epsilon$. Stable in this case means that the parameter is predictive on unseen data when the model is trained on reduced subset of parameters.

We apply model constraints on individual weights. Parameter weights $\hat{\theta }_{i,k}$ ( and $\theta _{i,m}$ in hierarchical SupirFactor), with *i* output and *k*, and *m* input features are removed if $\xi ^{2}_{i,k, L} \le \epsilon$ for the respective layer *L*, with a choice of $\epsilon = 0$, i.e., not predictive of the output, is the most conservative.

To enforce these constraints in number of regulators per gene, we use a model selection step after an initial training run (Fig. 1E). The model selection step is derived from $\xi ^{2}$ where

12 $$\begin{aligned} \widehat{\xi ^{2}}_{i, k} \equiv \frac{\xi ^{2}_{i, k}}{\overline{\xi ^{2}}_{i}} \end{aligned}$$

with $\overline{\xi ^{2}}_{i}$ as the maximum $\xi ^{2}$ for gene *i*. Selection is done iteratively by selecting a threshold $\epsilon$. The model is then refit with parameters where $\widehat{\xi ^{2}}_{i,k} > \epsilon$. This is done iteratively until convergence where at each step $\xi ^{2}$ is recomputed. $\epsilon$ is a measure of inclusion of relative predictive power. Using $\epsilon =0$ means all predictive links are kept after sub-setting and relative predictive power $\widehat{\xi ^{2}}$ is not impacting the subset.

Using $\widehat{\xi ^{2}}_{i, k}$ facilitate selecting regulator subsets where, unless the gene is too noisy or the prior lack sufficient information that can reliably predict the specific expression pattern (i.e., all $\xi ^{2}_{k} \le \epsilon$ for the gene in question), at least 1 TF can be inferred to be predictive relative to all *k* for that gene *i* and other regulators are ranked relative to it.

For the hierarchical model sub-setting, weights are eliminated if no predictive interactions can be derived from the indirect path between a latent feature *k* through the latent feature *m* in the subsequent layer to the set of joint output features, above the chosen $\epsilon$ threshold. If $\xi ^{2}_{k, m, \varvec{\Pi }} \le 0$ the hidden layer weight is pruned.

### Network component analysis (NCA)

Network component analysis (NCA) can be used to estimate TFA directly by formulating the causal network inference problem so that

13 $$\begin{aligned} \varvec{Y} = \varvec{\phi }\varvec{\Theta } \end{aligned}$$

where $\varvec{Y} \in \mathbb {R}^{S,n}$ is gene expression with *n* genes and *S* samples, $\varvec{\phi } \in \mathbb {R}^{m, k}$ is the TFA with *k* TFs, and $\varvec{\Theta } \in \mathbb {R}^{k, n}$ is the regulatory effects linking genes to TFs. The unknown “true” $\varvec{\Theta }$ is the regulatory interaction between genes and TFs we want to find. $\varvec{\phi }$ is unknown given the assumption that the expression level of a transcription factor *k* does not correlate well with the activity of the protein [66]. Therefore, we need to solve for both $\varvec{\Theta }$ and $\varvec{\phi }$, which forces us to convolve our estimation of regulatory effect and the TFA. To deconvolve and solve this, we impose a prior $\varvec{P}$ with elements $\in \{0, 1\}$ as an initial guess to the structure of $\varvec{\Theta }$ and use that to solve for an initial estimate for $\varvec{\phi }$.

14 $$\begin{aligned} \hat{\varvec{\phi }} = \underset{\varvec{\phi }}{\text {arg}\,\text {min}} \left\| {\varvec{Y} - \varvec{\phi }\varvec{P}}\right\| \end{aligned}$$

This is solved by ordinary least squares. The estimated $\varvec{\phi }$ is then used to solve for $\varvec{\Theta }$

15 $$\begin{aligned} \hat{\varvec{\Theta }} = \underset{\varvec{\Theta }}{\text {arg}\,\text {min}} \left\| {\varvec{Y} - \hat{\varvec{\phi }}\varvec{\Theta }}\right\| \end{aligned}$$

This is interpreted as an estimate of TFA given a number of reporter genes defined by the prior $\varvec{P}$, i.e., the expression level of the target genes is a proxy for how active a TF is in any given sample. The variance of the TF activity is defined and constrained by the variance of the reporter genes.

### Epigenetic masking

To incorporate the paired ATAC- and RNA-sequencing data we create a masking scheme to mask input gene expression profiles with the ATAC so that expression input

16 $$\begin{aligned} \dot{x}_{i, s} = x_{i, s} \cdot 1~ \text{ if }~ a_{p, s} \in A_{i}~ \text{ else }~ 0 \end{aligned}$$

with $x_{i, s}$ being the expression of gene *i* in sample *s* and $a_{p, s}$ being an available ATAC peak *p* in the set of accessible regions $A_{i}$ associated with gene *i*.

### Data acquisition

#### Bulk expression data

*Bacillus subtilis* bulk expression data for data set 1 (B1) [10] and data set 2 (B2) [67] and the known prior network [10] were used as previously described [41]. *Saccharomyces cerevisiae* bulk expression data for data set 1 [68] and data set 2 [69], and the known prior network [70] were used as previously described [41].

#### Single cell expression data

*Saccharomyces cerevisiae* single cell training data was assembled from [13] and [71] as previously described [41].

*S. cerevisiae* test data was collected using a method previously published [13]. In short, biological replicates containing unique, transcriptionally expressed molecular barcodes of a wild-type strain 1 (*MAT*$\alpha$*/MATa* $\Delta$*ho::NatMX/*$\Delta$*ho::KanMX*) and a wild-type strain 2 (*MAT*$\alpha$*/MATa HO/*$\Delta$*ho::NatMX HAP1+::pACT1-Z3EV::NatMX/HAP1 ura3*$\Delta$*0/URA3 can1*$\Delta$*::prSTE2-HIS5/CAN1 HIS3/his3*$\Delta$*1 LYP1/lyp1*$\Delta$*0*) were generated as previously described [13].

Strains were grown overnight in rich media (YPD as previously described [13]) and then subcultured into 100 mL YPD or minimal media (MMD as previously described [13]) for 3 h. Cells from each flask were then taken, fixed with saturated ammonium sulfate, processed, and sequenced using the protocol as previously described [13]. Raw sequencing data was processed into count data using a previously-described pipeline [13] which joined the transcriptional barcodes to individual cells, assigning specific genotypes to cells and removing any cell containing multiple distinct barcodes as doublets. Four data sets were then created from this count table; YPD 1 (*n *= 1531, wild-type strain 1 in rich YPD media), YPD 2 (*n *= 1428, wild-type strain 2 in rich YPD media), MMD 1 (*n *= 492, wild-type strain 1 in minimal MMD media), and MMD 2 (*n *= 463, wild-type strain 2 in minimal MMD media). This data is deposited in NCBI GEO as GSE218089.

#### Yeast test GRN

Yeast network performance evaluation was performed using a previously described high-confidence gold-standard GRN [41, 68] containing 1403 regulatory edges connecting 993 genes to 98 TFs. The yeast prior knowledge network consists of 11,486 regulatory edges connecting 3912 genes to 152 TFs, and many of the edges are shared with the gold-standard GRN, necessitating the hold-out for testing strategy used for performance evaluation.

An additional test GRN was constructed which had no edges from the prior knowledge network. This test GRN was obtained from the Saccharomyces Genome Database (yeastgenome.org) and consists of 29,253 regulatory edges connecting 6367 genes to 170 regulatory TFs. We further modify it by masking every regulatory edge which occurs in the prior knowledge network, excluding it from scoring. This yields a test GRN with 25,621 edges, none of which occur in the prior knowledge network or the gold-standard GRN, connecting 6367 genes to 170 regulatory TFs.

#### Yeast annotations

Cell cycle related yeast genes are annotated based on [72]. Ribosomal, ribosomal biogenesis, and induced environmental stress response genes are annotated based on [73].

Individual cells in single cell RNA expression data sets are assigned a cell cycle phase based on cell cycle gene annotations. Expression of each cell cycle gene is normalized to a mean of 0 and unit variance. All marker genes annotated with a specific cell cycle phase (G1, S, G2, M, or M/G1) are grouped, and the cell is assigned to the phase that has the maximum mean group expression.

Kyoto Encyclopedia of Genes and Genomes (KEGG) annotations [47] were selected to cover the majority of the core yeast metabolism. KEGG annotations are KEGG:04111 (Cell cycle), KEGG:00010 (Glycolysis), KEGG:00020 (TCA cycle), KEGG:00500 (Starches), KEGG:00660 (C5-Branched), KEGG:01210 (C5-Branched), KEGG:00250 (AA (A, D, E, N, Q)), KEGG:00260 (AA (G, S, T)), KEGG:00330 (AA (R & P)), KEGG:00400 (AA (F, Y, W)), KEGG:00270 (AA (C & M)), KEGG:00920 (Sulfur), KEGG:00061 (Fatty acids), KEGG:00230 (Purines), and KEGG:00240 (Pyrimidines).

#### PBMC multi-ome dataset preprocessing

Paired PBMC scRNA-seq and scATAC-seq was downloaded from the 10x website [74]. This data was preprocessed using a previously published workflow [48]. In short, the RNA-seq data is preprocessed as detailed in the “Single cell pre-processing” section, with the additional filtering of cells with $>25000$ or $<1000$ counts and $<20\%$ mitochondrial counts of total. For the ATACseq data, we used epiScanpy [75], filtering peaks in $<10$ cells and cells with $< 5000$ or $>7\cdot 10^{4}$ counts, and with a variability score $<0.515$. Final data contains 10,411 cells, 21,601 genes, and 75,111 peaks.

Cell types are annotated using the reference PBMC dataset [59] passed to scanpy’s [76] inject label transfer function, resulting in 8 annotated celltypes (Fig. 5A).

#### ENCODE PBMC prior knowledge network construction

TF-ChIP peaks were obtained as narrowPeak BED files from the ENCODE project database. The GRCh38 genomic annotations (NCBI GCF_000001405.39) were obtained as a GTF file from NCBI and filtered for protein-coding genes.

TF-ChIP peaks were linked to candidate target genes with the inferelator-prior package [41]. TF peaks were annotated as possible regulators of a gene if they were within 50 kbp upstream of a gene transcription start site and 2 kbp downstream of a gene transcription site, with no other gene between the TF peak and the gene transcription start site. TF peaks were further filtered to remove any regulators not annotated as TFs (including GTFs, chromatin modifiers, and polymerase subunits).

This large pool of potential TF regulatory peaks were then subset for intersection with annotated regulatory regions for PBMC cell types (ENCODE Accession IDs ENCFF776AJJ, ENCFF497NXM, ENCFF984SPH, ENCFF079TQT, ENCFF862ULW, ENCFF504FDC, and ENCFF905BHJ). The peak intensity (signalValue) was summed for all peaks annotated to each TF-gene pair to generate genes by TFs putative regulatory matrix. This matrix was further constrained for sparsity by retaining at most 1.5% non-zero values for each TF, shrinking all values below this threshold to zero, and producing a genes by TFs prior knowledge network matrix with a sparsity of 1.22%.

#### PBMC COVID dataset preprocess

Healthy and COVID patient single cell data from [58] was fetched from [77]. Expression counts were preprocessed similarly as the PBMC data (see the “PBMC multi-ome dataset preprocessing” section).

### Data preprocessing and model parameterization

#### Single cell pre-processing

For the single cell data, unless otherwise stated, we follow standard normalization procedures which include (i) filtering genes with expression in $< 10$ cells and (ii) count normalization, scaling each cells total count to the same value over the dataset. This serves to eliminate the effect of variable sequencing depth in the experimental technique, and (iii) log transforming the (data + 1) using the natural logarithm.

#### Feature normalization

For bulk data, we use the standard normalization of each input feature so that

$$\begin{aligned} z = \frac{x-\mu }{\sigma } \end{aligned}$$

for each gene with $\mu =$ mean and $\sigma =$ standard deviation over the gene.

To preserve the sparse structure of single cell data, we, in addition to the above, adopt a robust normalization approach without centering. Each gene is scaled by the range of the 1 and 99 percentile and shifted so the lowest value for each gene $=0$ implemented using the scikit-learn RobustScaler method [78] which we call RobustMinScaler.

#### ReLU in hierarchical SupirFactor

For DNN linear activation does not contribute meaningfully in different layers and can be reduced to a single linear map. The rectified linear unit (ReLU) [44] truncates activation to stay strictly positive and injects non-linearities into the model architecture. For hierarchical SupirFactor, we therefore use ReLU and define the gradient for the ReLU function

$$\begin{aligned} \text{ ReLU}_{0} \equiv \sigma (z) = \max {(0, z)} \end{aligned}$$

so that with $z = 0$ the gradient is $\equiv 1$. With *z* as the linear combination of inputs to each feature.

#### Data preprocessing and model parameterisation

Visualizations throughout this work, if not stated otherwise, were generated in Matplotlib [79] with some components done with seaborn [80] and scanpy [76].

### Supplementary information

**Additional file 1.** Supplementary information. This file contains all supplemental figures along with their descriptions.

**Additional file 2.** Peer review history.

## Data Availability

The most recent iteration of the open-source SupirFactor software, released under the permissive BSD 2-Clause “Simplified” License, is available at https://gitlab.com/Xparx/supirfactor [81]. The specific version of the software used in this publication is accessible on Zenodo through the following link: https://zenodo.org/doi/10.5281/zenodo.10161545 [82]. The Zenodo repository also contains the trained models used in the manuscript for both single-cell yeast (scY) and peripheral blood mononuclear cell (PBMC) datasets. Additionally, the repository encompasses the inferred gene regulatory networks (GRNs) between transcription factors (TFs) and genes, and meta TFs and genes. Furthermore, it contains the TF activity (TFA) and mTFA for each model for the training data. All publicly available and previously published data referenced and used in the manuscript are detailed in the “Data acquisition” section, with the repository on Zenodo containing the used versions of priors, gold standards, and bulk expression benchmark datasets. Regarding the single-cell data. *S. cerevisiae* was downloaded from the NCBI GEO database. The training data originated from GSE144820 and GSE125162, while the validation data came from GSE218089. PBMC data was obtained from https://support.10xgenomics.com/single-cell-multiome-atac-gex/datasets/1.0.0/pbmc_granulocyte_sorted_10k [74], and the additional PBMC COVID dataset was acquired from https://ndownloader.figshare.com/files/27458837 [77].
