# Supplementary material for: Structure-primed embedding on the transcription factor manifold enables transparent model architectures for gene regulatory network and latent activity inference
Source: Genome Biol. 2024 Jan 18;25:24. doi: 10.1186/s13059-023-03134-1 (PMC10797903; doi:10.1186/s13059-023-03134-1)
Supplement: Supplementary file 1 — Additional file 1. Supplementary information. This file contains all supplemental figures along with their descriptions. [file 13059_2023_3134_MOESM1_ESM.pdf]

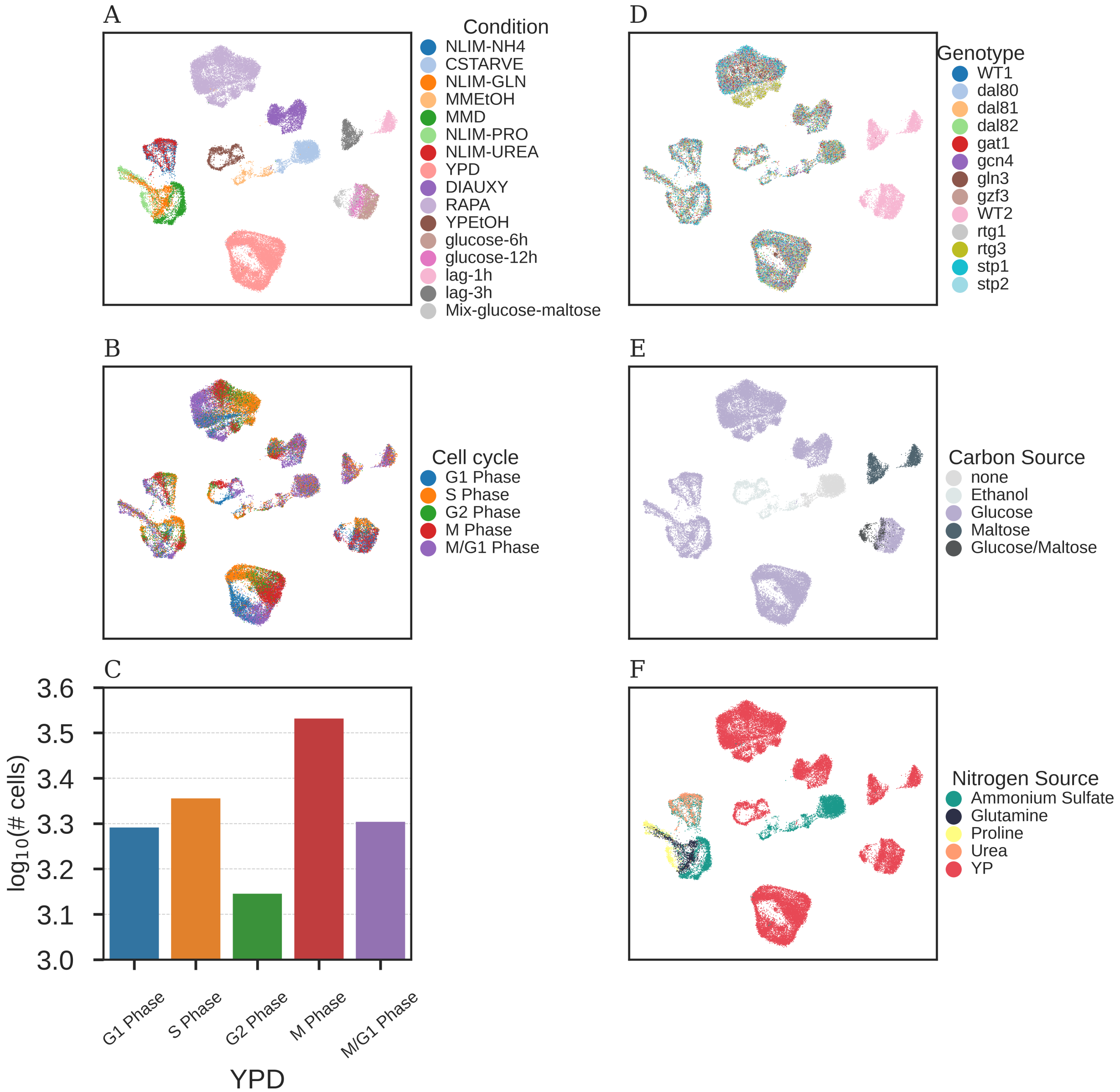

Fig S1: Overview of the single cell combined datasets [13] and [82] (scY) in *S. cerevisiae*. UMAP [83] projected data with annotation of **A**: growth condition, **B**: inferred cell cycle phase, and **C**: Frequency of cell cycle annotated cells in YPD. **D**: Genotypes. **E**: Carbon source for growth condition. **F**: Nitrogen source for growth condition.

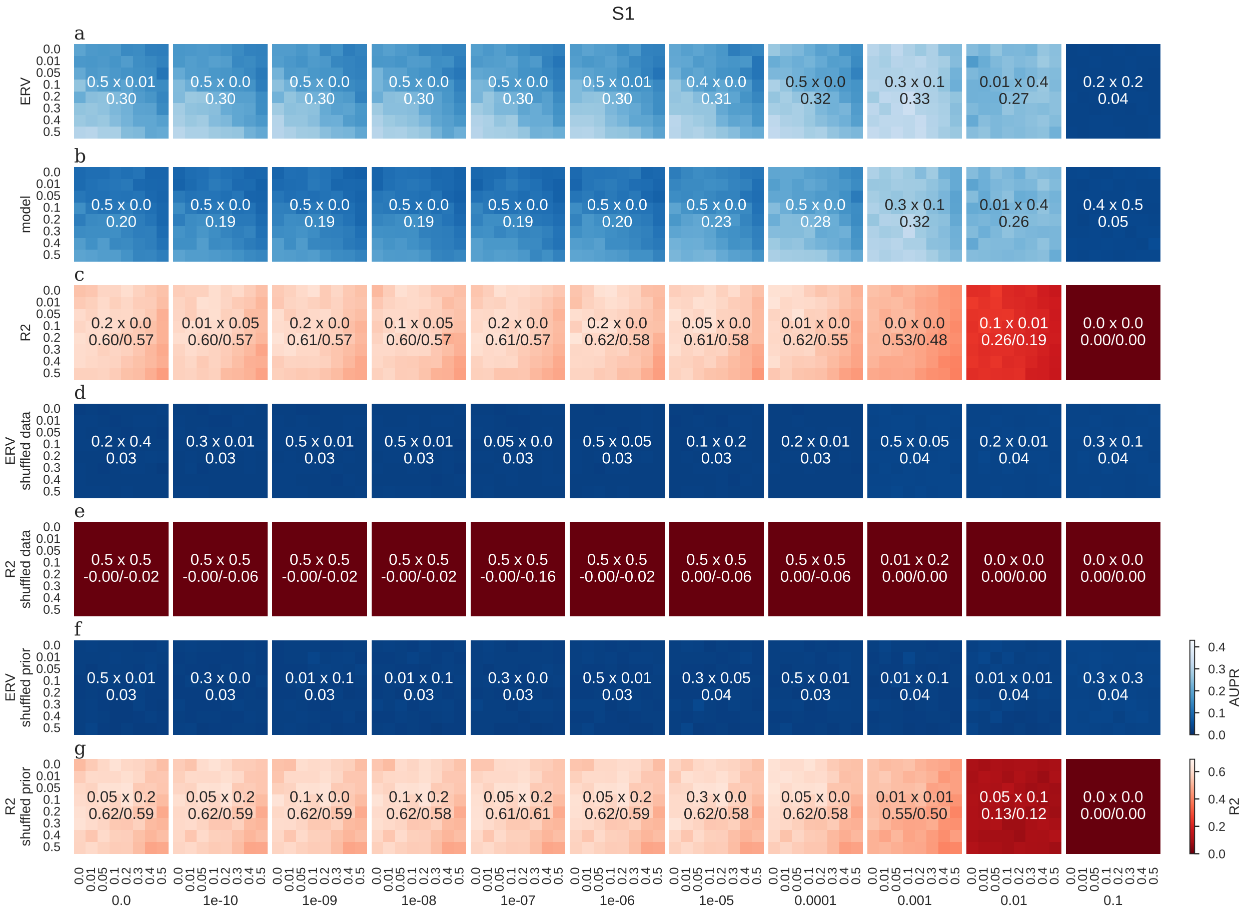

Fig S2: Dataset S1[70]. Hyper-parameter cube search. Each column of squares indicate a weight-decay (L2) penalty, with 0 and then 10 log linear values between $10^{-10} - 10^{-1}$. Each square is the grid search of dropout on input feature (y-axis) and latent features (x-axis). Rows in blue shade (**a**, **b**, **d** and **f**) indicate GRN recovery performance measured by area under precision recall curve (AUPR) on 20% held out of gold-standard network. ERV (**a**, **d** and **f**) indicate the GRN is ranking based on explained relative variance (Section), model (**b**) indicate network interactions are ranked based on their weights magnitude, with larger magnitude indicate more influential. Red shades (**c**, **e** and **g**) indicate prediction accuracy R^2 on held out, n=50%, of samples. Negative controls are done by shuffling either the data (**d** and **e**) or the prior network structure (**f** and **g**).

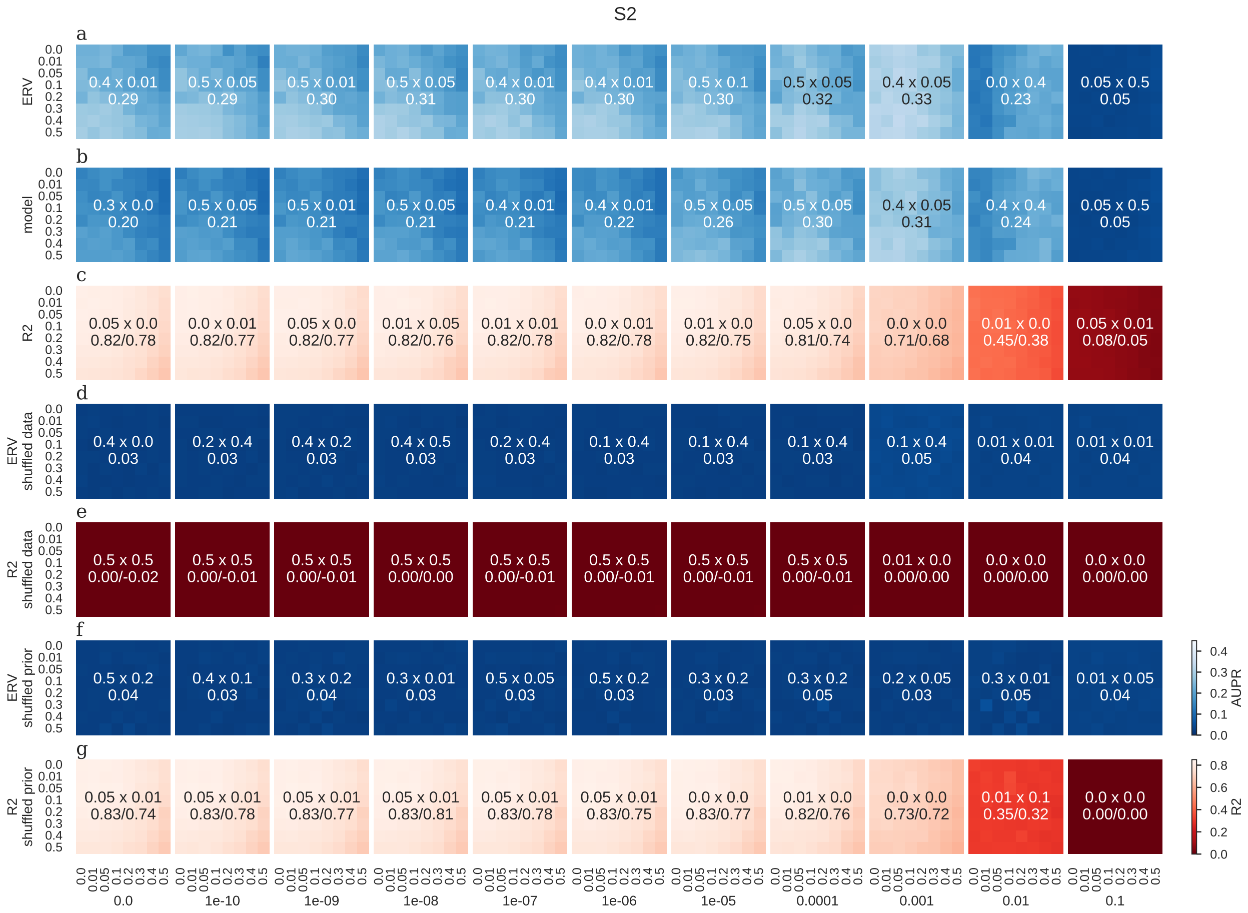

Fig S3: Dataset S2[69]. Description as in Figure Fig S2

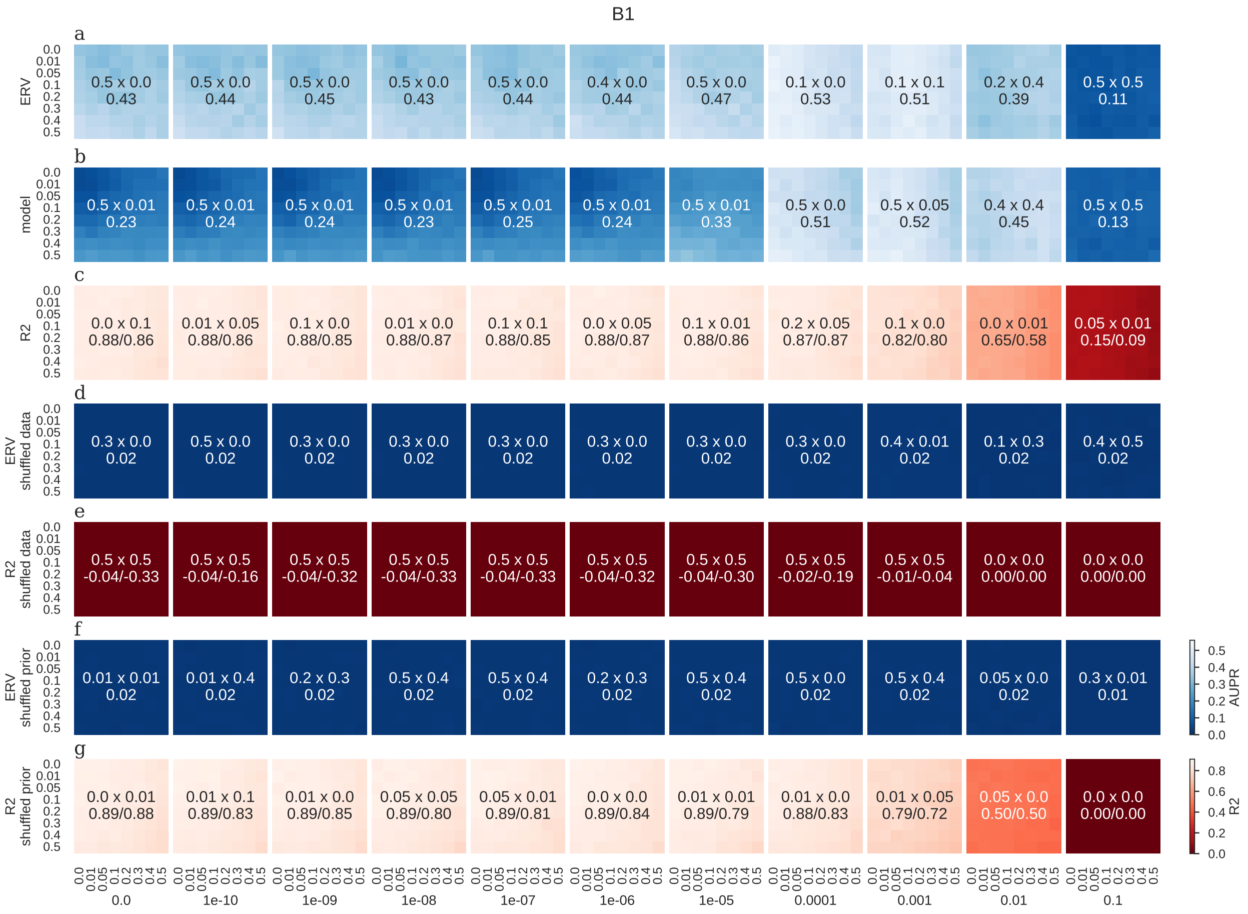

Fig S4: Dataset B1[10]. Description as in Figure Fig S2

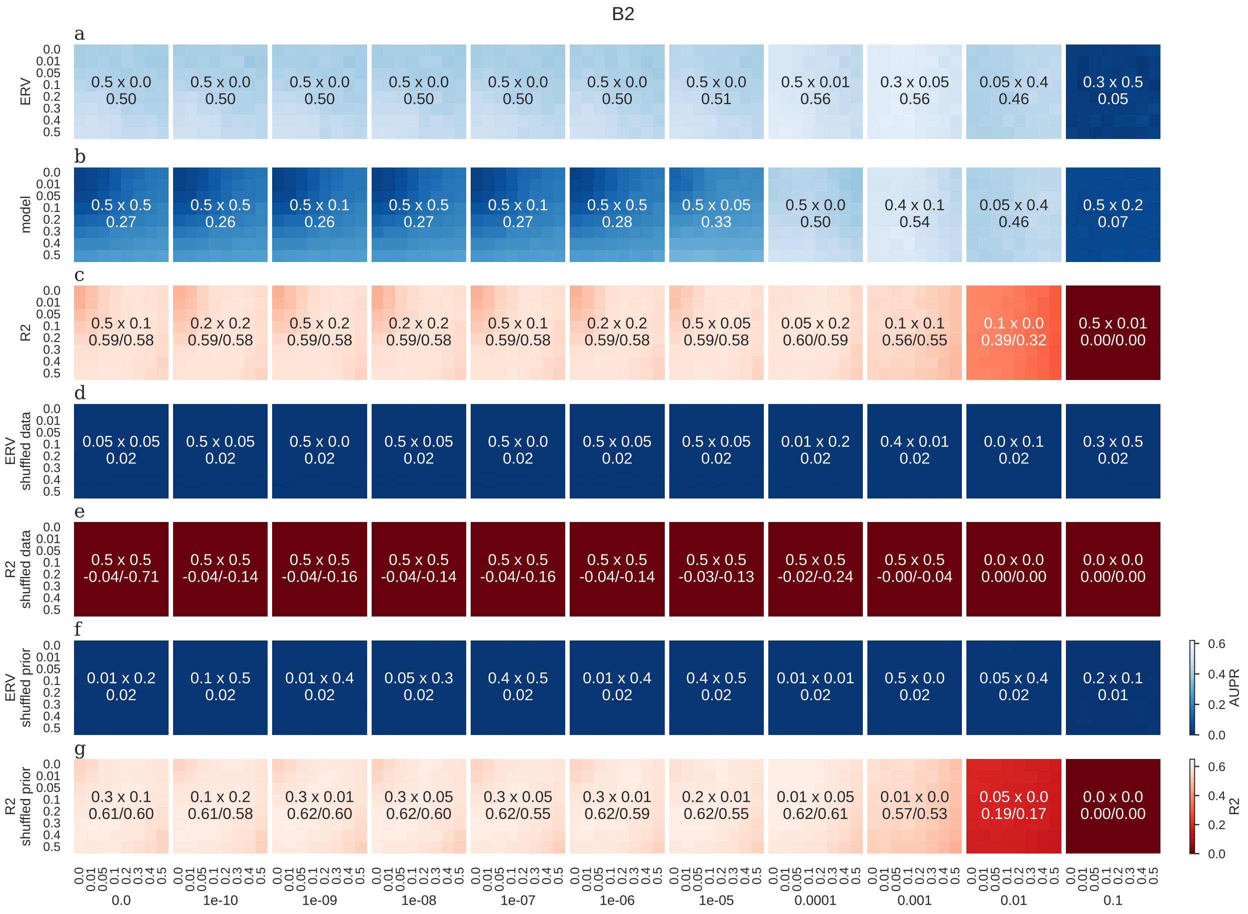

Fig S5: Dataset B2[68]. Description as in Figure Fig S2

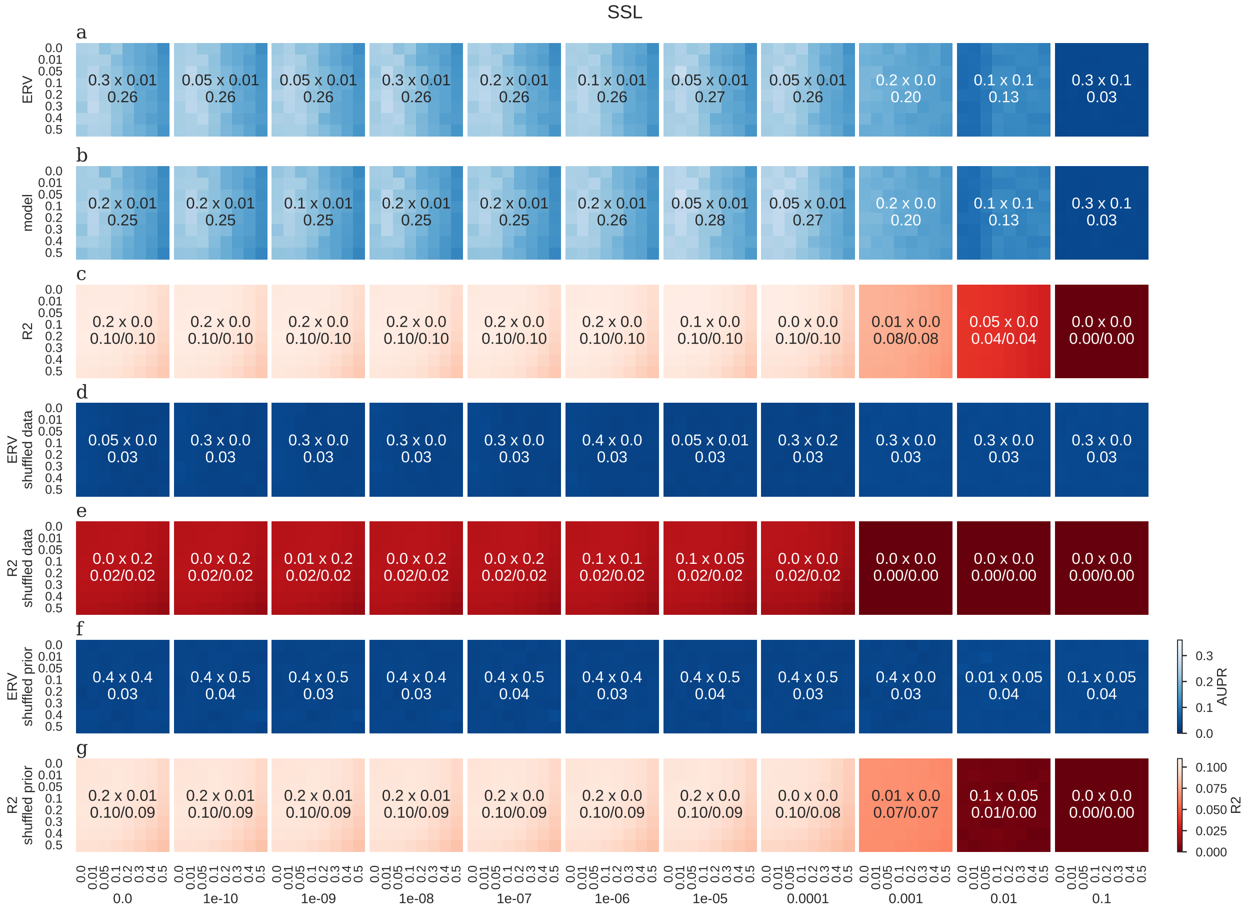

Fig S6: Dataset scY[13, 82] with StandardScaler normalisation and linear activation (SSL) configuration. Description as in Figure Fig S2

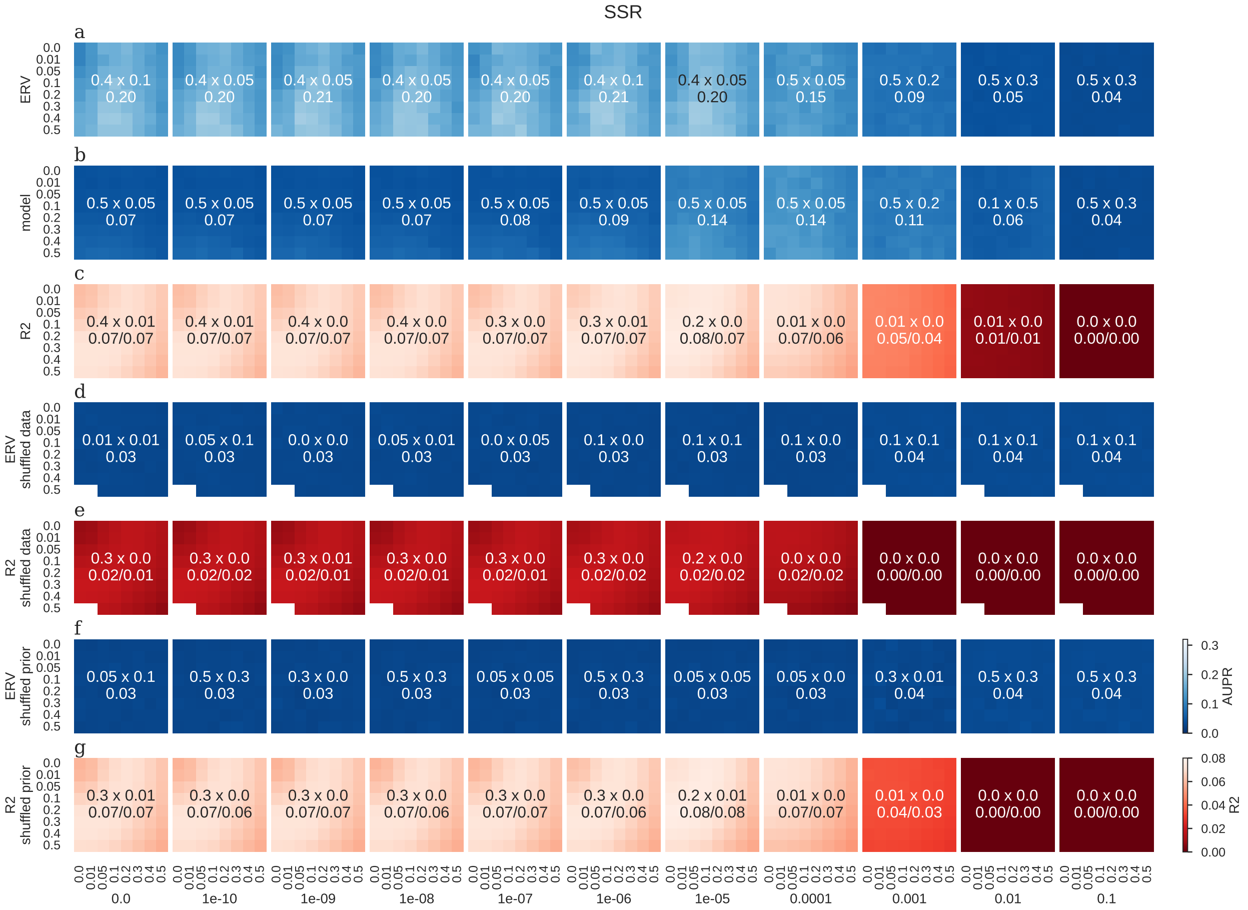

Fig S7: Dataset scY[13, 82] with StandardScaler normalisation ReLU_0 activation (SSR). Description as in Figure Fig S2

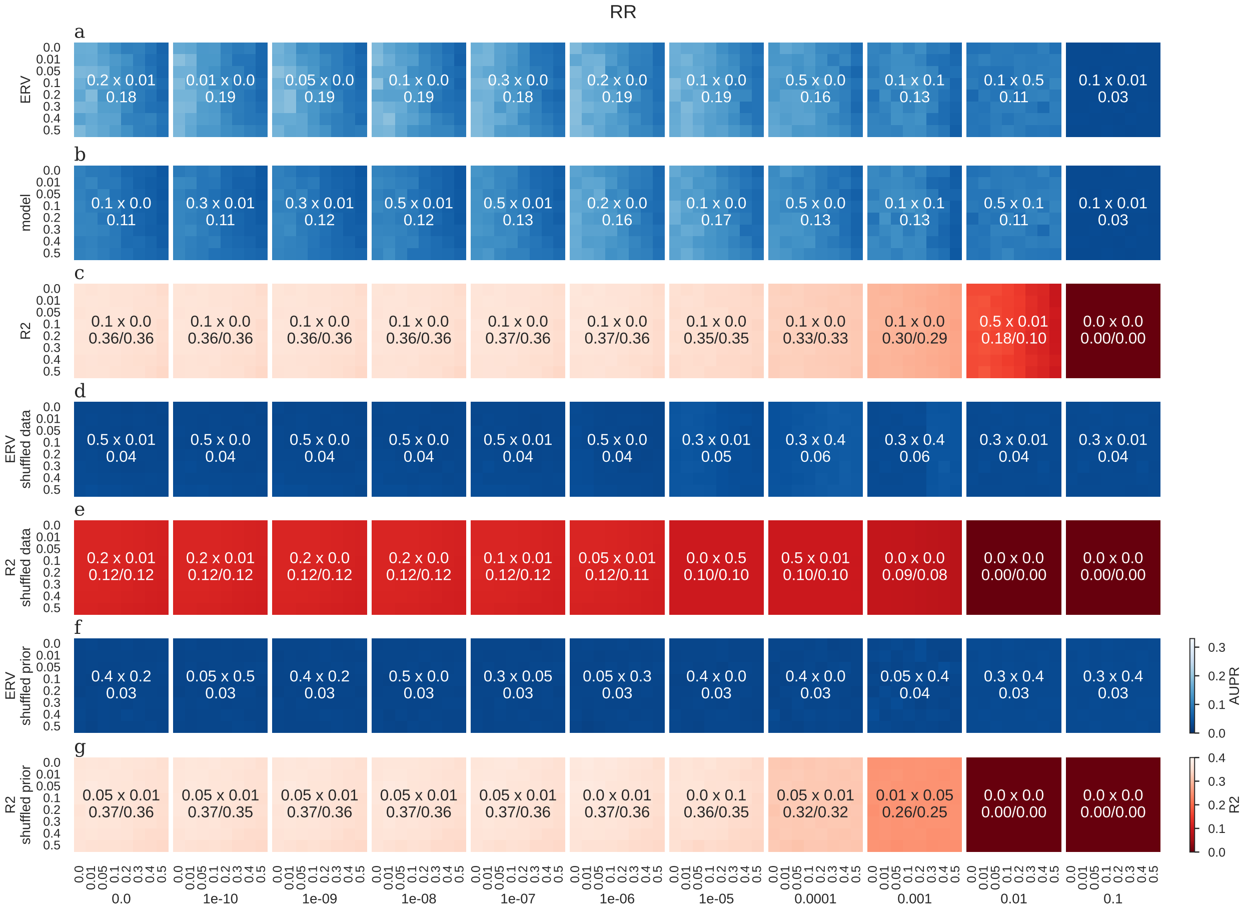

Fig S8: Dataset scY[13, 82] with RobustMinScaler normalisation and $ReLU_0$ activation (RR). Description as in Figure Fig S2

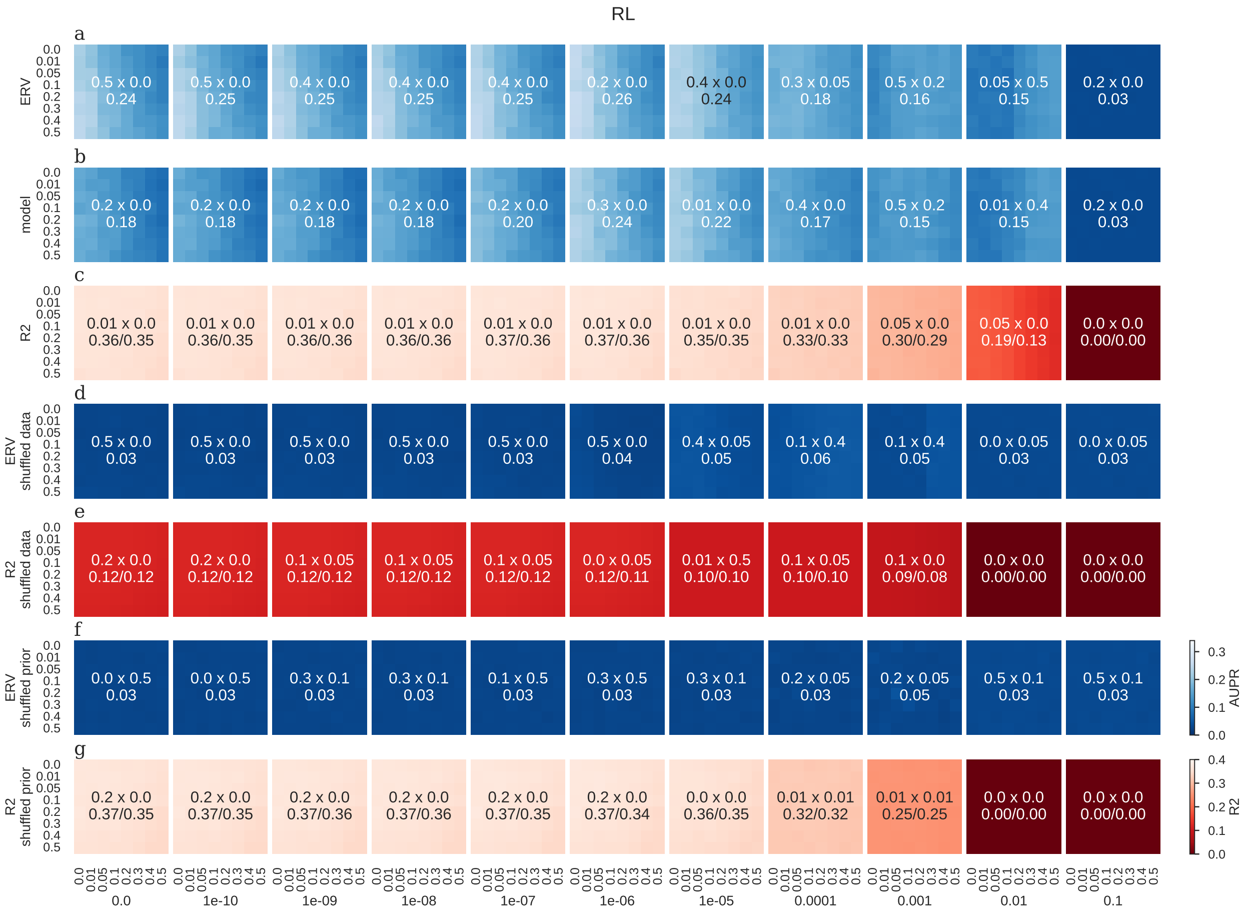

Fig S9: Dataset scY[13, 82] with RobustMinScaler normalisation and linear activation (RL). Description as in Figure Fig S2

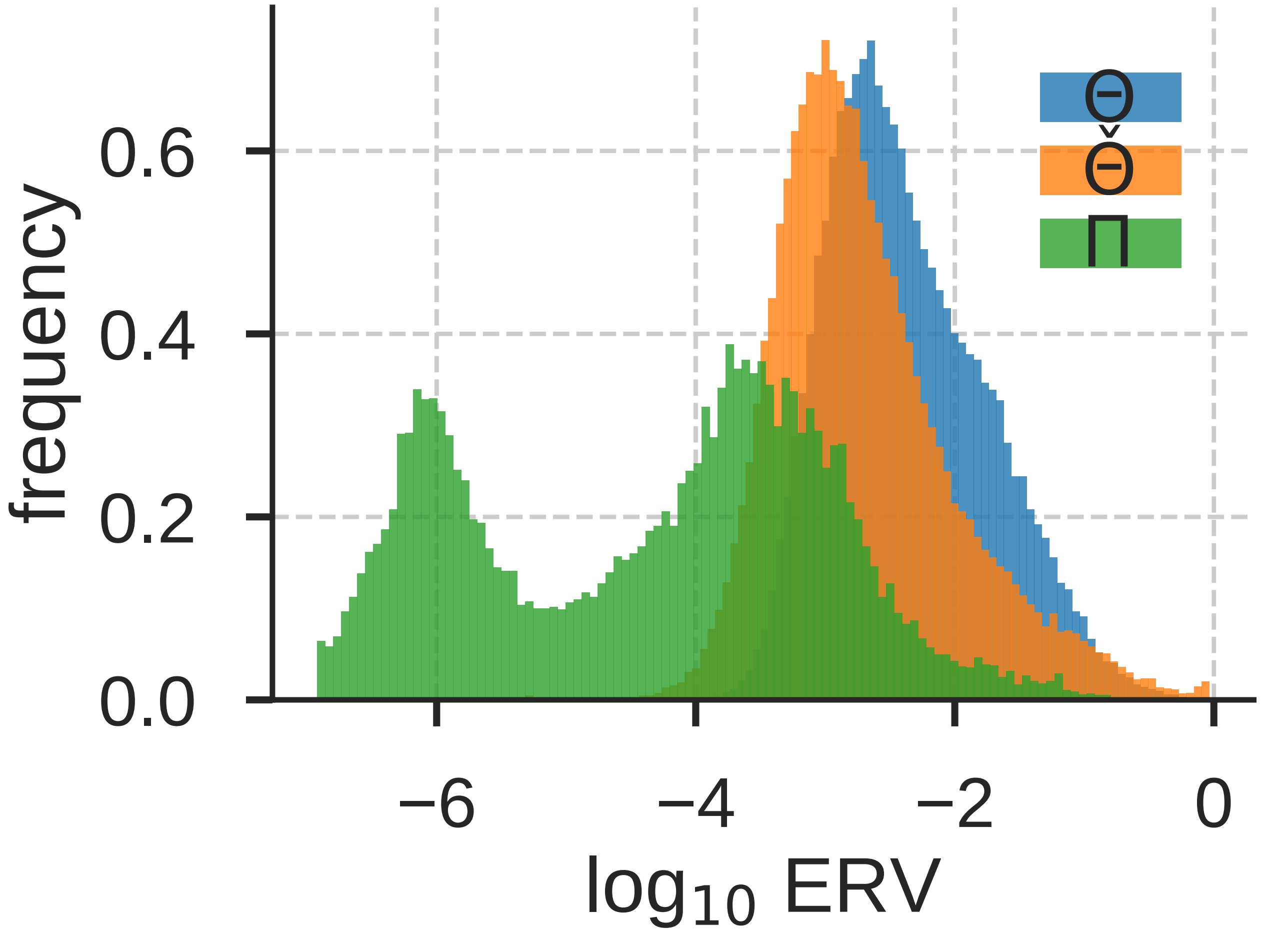

Fig S10: Distribution of ERV (ξ^2) for hierarchical SupirFactor trained on scY.

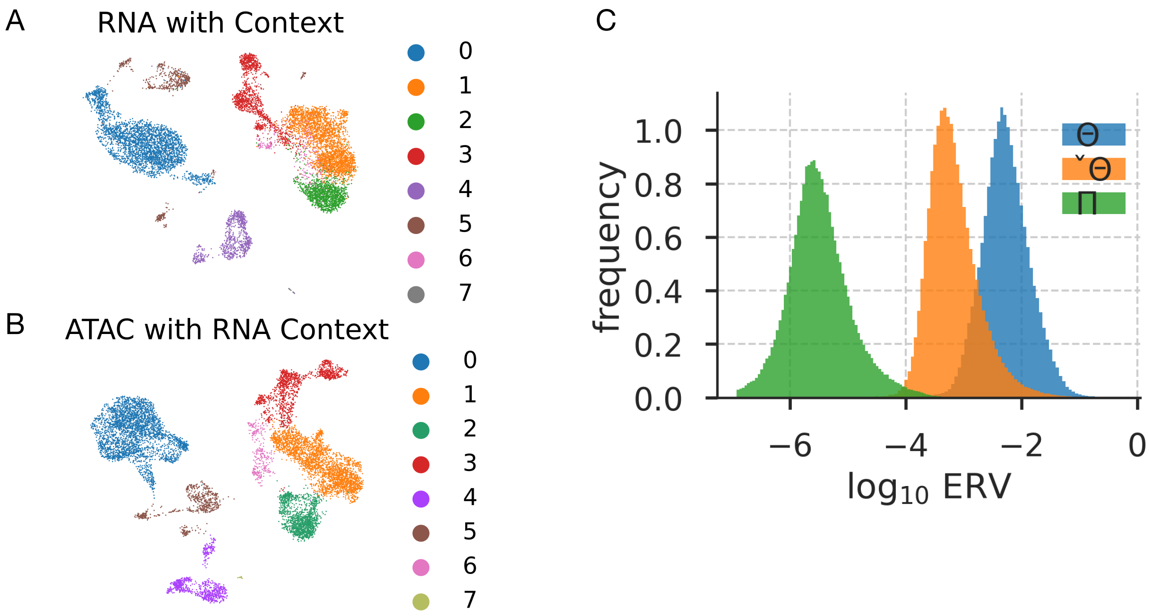

Fig S11: Single cell PBMC Contexts and Networks **A**: UMAP projection of the scRNA-Seq PBMC dataset with "Context" via leiden clustering (0.2) **B**: UMAP projection of the scATAC-Seq PBMC dataset with "Context" via leiden clustering (0.2) **C**: Distribution of ERV for $\hat{\boldsymbol{\Theta}}$, $\boldsymbol{\Pi}$ and $\boldsymbol{\Theta}$ networks

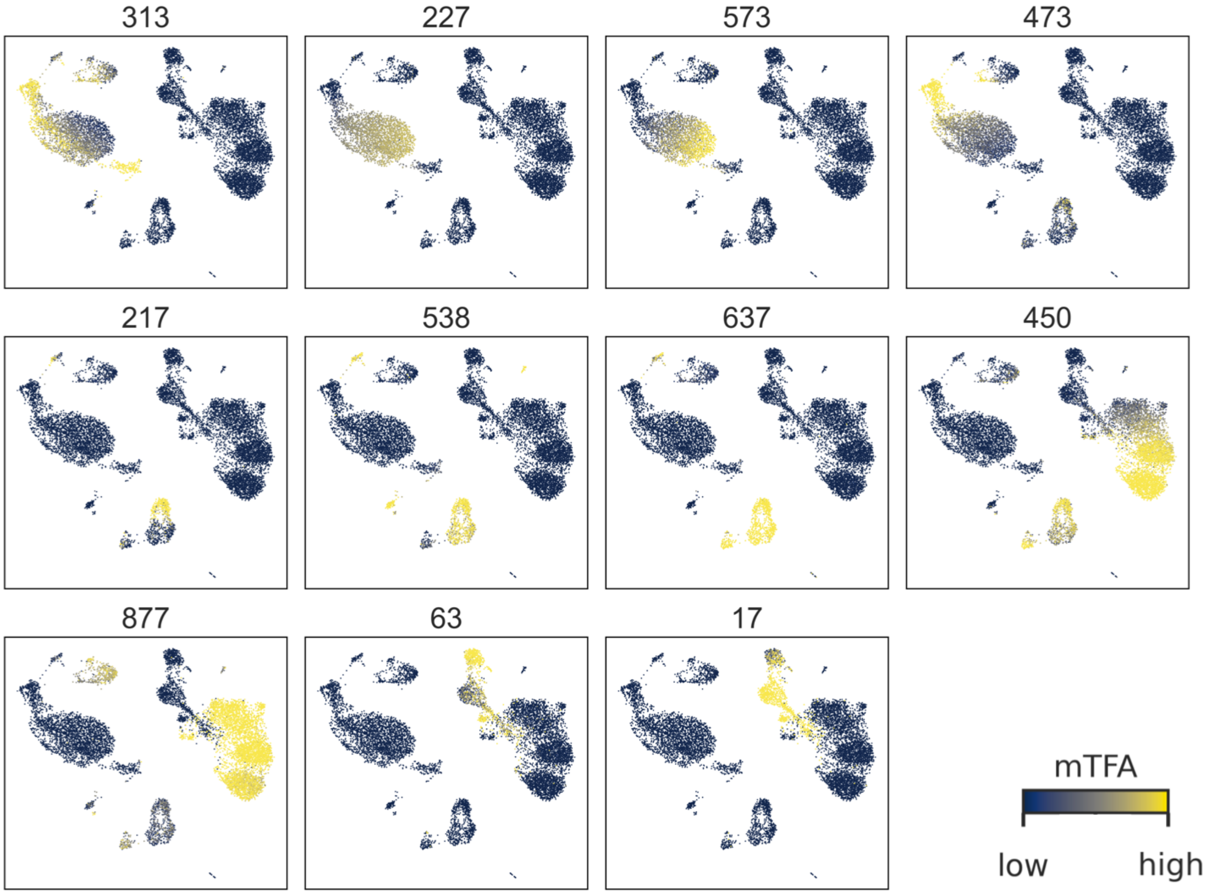

Fig S12: UMAP projection of all functionally enriched mTF activation (mTF activity scaled [0, 1] for comparison).

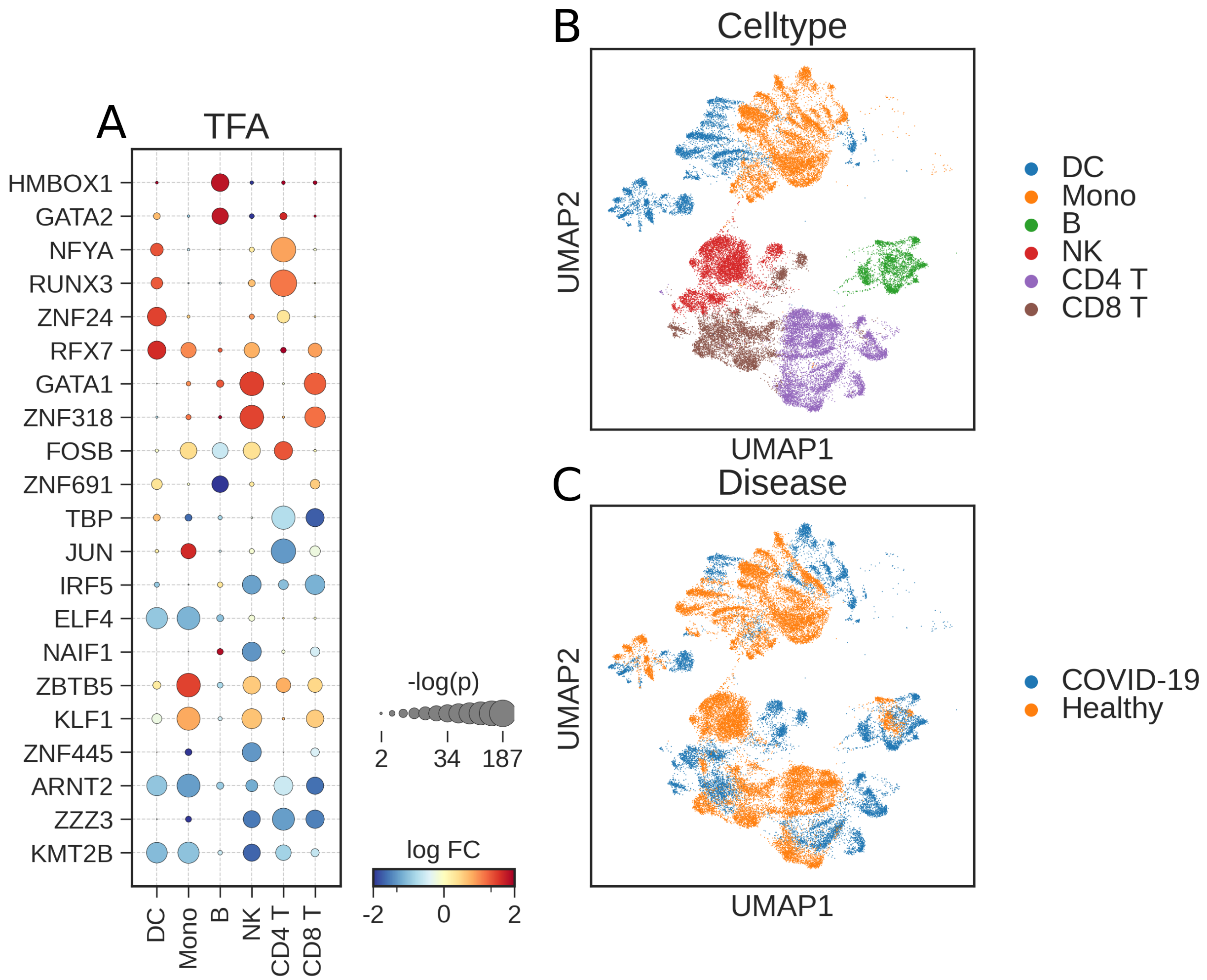

Fig S13: Transcription factor activity (TFA) analysis on [59], fetched and pre-processed as described in Section . **A**: Differently active TFs was computed for each cell type, comparing Healthy vs Disease, using t-test with Bonnferroni correction from the scanpy package. From that we pick the top 2 TFs active in the disease and top 2 TFs in healthy for each test condition, log transformed to $-\log(p)$. **B**: Celltype labels were recovered from the predefined labels in *final_clust_v2* in the data where CDC1, CDC2 and PDC was merged into DC, and MC MONO, C MONO_1 and C MONO_2 into Mono for our analysis. **C**: COVID-19 and Healthy annotation.
