# Supplementary material for: Structure-primed embedding on the transcription factor manifold enables transparent model architectures for gene regulatory network and latent activity inference
Source: Genome Biol. 2024 Jan 18;25:24. doi: 10.1186/s13059-023-03134-1 (PMC10797903; doi:10.1186/s13059-023-03134-1)
Supplement: Supplementary file 2 — Additional file 2. Peer review history. [file 13059_2023_3134_MOESM2_ESM.docx]

**Review history**

**First round of review**

**Reviewer 1**

Gene regulatory network (GRN) modeling has become an indispensable tool in recent years, providing valuable insights into the intricate interactions that underlie gene expression and cellular function. Andreas Tjärnberg and colleagues address current limitations in GRN inference methods, which arise from the lack of direct measurements of regulatory features in genome-wide screens. As a result, these methods often depend on gene expression data as a proxy for upstream independent features, complicating the validation and prediction of modeling frameworks.
To address these challenges, the authors propose a novel deep learning autoencoder-based framework called StrUcture Primed Inference of Regulation using latent Factor ACTivity (SupirFactor). This framework scales to single-cell genomic data while retaining interpretability and integrates transcription factor (TF) binding and target gene expression data to estimate TF activity (TFA) as a latent feature, concurrently performing GRN inference. By accounting for non-linearities such as saturation and TF-TF interactions, SupirFactor provides a more accurate representation of biological systems.
The most notable advantage of SupirFactor over existing methods is its ability to maintain interpretability, which is often compromised in deep learning frameworks due to increased model complexity. This study demonstrates that SupirFactor outperforms leading GRN inference methods, predicts biologically relevant TFA, and elucidates functional regulatory pathways through the aggregation of TFs.
Tjärnberg et al.'s work represents a significant contribution to the field of GRN inference, offering a powerful and transparent framework for researchers studying complex biological systems. The paper also presents robust statistical analyses to demonstrate the performance of SupirFactor. While I am generally positive about the paper, I have some critiques and suggestions for improvement:
In the introduction (row 49-50), the statement about techniques being unsuitable for model pruning or selection is unclear. It may be more helpful to mention methods that can utilize prior knowledge for model selection and discuss the importance of metric boundedness.
I appreciate the benchmarking against GRN datasets and the detailed discussion of unique predictions. However, it would be interesting to see a more statistical analysis of TF activities, perhaps by examining correlations between TFA estimations in disease datasets and disease associations of the underlying TFs. This could further strengthen the method's applicability to genome medicine applications.
It would be intriguing to explore the impact of documented TF interactions on the learning process. For example, using protein-protein interaction (PPI) data as a potential prior for estimating duplex activities.
Some minor comments:
a. Page 5, line 39: The notation should be corrected to L2 ∈ 10^−6 − 10^−4.
b. Page 10, line 38 (Fig. 5B): Clarification is needed regarding mTF 227, as the gene ratio for associated genes related to the Phagosome pathway appears minimal (<0.01?), and the P-value is too small to discern.
The page numbers refer to the manuscript's page numbers.
Overall, this work represents a vital advancement in GRN inference, and with some additional clarification and analysis, it can further strengthen its impact on the field.

**Reviewer 2**

In the manuscript authored by Tjarnberg et al, a novel GRN prediction method, SupirFactor, is proposed. SupirFactor uses target gene expressions and regulatory links and functional pathway information as prior information to learn gene regulatory networks (GRN) and TF activities (TFA) by autoencoder. The authors have performed various benchmarking experiments to evaluate the prediction accuracy of SupirFactor and functional interpretations of SupirFactor results. This method provides a new way of quantifying TF activities from expression data. However, the TFA itself is not a clearly defined concept (see below my comments). The importance of TFA therefore remains unclear to me with respect to what part of regulatory activity (transcription, splicing, translation, TF binding kinetics) it may help to give better interpretation than other methods. I would like to invite the authors to consider the following comments.

Major
1. One central question for TFA is how it is defined. It is fine to claim that TFA is difficult to measure experimentally. But it is important to define what it represents. Does it represent abundance of nascent mRNAs or any types of transcription/splicing/translation/binding kinetics for TFs? There should be some discussions or analyses to spell out its biological relevance.
2. I think the biggest concern is that the ability to reconstruct target gene expressions does not necessarily indicate the true regulatory activities. The success of reconstruction may merely mean the model can learn how to remove the arbitrary noise from prior TF-target information, which may be the systematic bias within one dataset. In this case, holding-out part of the gold-standard GRN can give you good results but the inferred GRN might not be well generalizable. Other than hold-out validation using gold-standard GRNs, further experimental evidence(s) is needed. Such experimental evidence should be totally independent of the prior information used in generating the GRNs.
3. Although the authors claimed that TF expressions do not truly reflect TF activities. I agree with this. However, one common (and simple) way to construct GRN is still to train a model to predict target expressions using TF expressions. The benchmark experiment may need to include at least one of these methods to show SupirFactor's advantage (e.g. GENIE3, GRNboost2).
4. The choice of informative genes as inputs is important for modeling. But the manuscript does not discuss how informative genes were selected. How were informative genes selected so as to reflect TFAs? This should be discussed.

Minor
1. Is the inferred GRN only a subset of the prior GRN? The training process will iteratively prune the links that does not have good xi^2 scores. Are these removed links all from original prior GRN or SupirFactor can prune links not present in the prior GRN? Can this method discover new links not in the prior GRN?
2. In 2.3, the authors stated that max R2 improves the R2 at a cost of decreasing AUPR, but max R2 actually has higher AUPRs in fig 2A and fig 2B.
3. The authors showed that RobustMinScaler outperformed StandardScaller and sparsity of RobustMinScaler gave the advantage. This is interesting. Can this be caused by the fact that standardization is unbound? The zeros of each gene got transformed into different negative values then this can confuse the model, introducing technical noise.
4. Why testing R^2 is higher than training R^2 in fig. 2E?
5. In section 2.5 the authors stated that " and we see that these conditions have very similar mTF activation (Figure 4B)". The authors should specify the exact groups they referred to.
6. In 4.3.1, the penalty strength is set to be identical to W, theta and PI. What if the three parts are penalized differently?
7. More details should be given to mTF layer. Does mTF layer have the same number of neurons as TF layer? Also details about number of neurons in each layer should be specified.

**Authors’ response to reviewers**

Reviewer #1:

Gene regulatory network (GRN) modeling has become an indispensable tool in recent years, providing valuable insights into the intricate interactions that underlie gene expression and cellular function. Andreas Tjärnberg and colleagues address current limitations in GRN inference methods, which arise from the lack of direct measurements of regulatory features in genome-wide screens. As a result, these methods often depend on gene expression data as a proxy for upstream independent features, complicating the validation and prediction of modeling frameworks.
To address these challenges, the authors propose a novel deep learning autoencoder-based framework called StrUcture Primed Inference of Regulation using latent Factor ACTivity (SupirFactor). This framework scales to single-cell genomic data while retaining interpretability and integrates transcription factor (TF) binding and target gene expression data to estimate TF activity (TFA) as a latent feature, concurrently performing GRN inference. By accounting for non-linearities such as saturation and TF-TF interactions, SupirFactor provides a more accurate representation of biological systems.
The most notable advantage of SupirFactor over existing methods is its ability to maintain interpretability, which is often compromised in deep learning frameworks due to increased model complexity. This study demonstrates that SupirFactor outperforms leading GRN inference methods, predicts biologically relevant TFA, and elucidates functional regulatory pathways through the aggregation of TFs.
Tjärnberg et al.'s work represents a significant contribution to the field of GRN inference, offering a powerful and transparent framework for researchers studying complex biological systems. The paper also presents robust statistical analyses to demonstrate the performance of SupirFactor.


While I am generally positive about the paper, I have some critiques and suggestions for improvement:

In the introduction (row 49-50), the statement about techniques being unsuitable for model pruning or selection is unclear. It may be more helpful to mention methods that can utilize prior knowledge for model selection and discuss the importance of metric boundedness.

The original draft was unclear on these points. We have restructured the paragraphs and focused on examples of knowledge priming models and metric bounds in relation to other methods for model interpretation. The last two paragraphs of the introduction now read as follows: :

Using more complex models to better match known transcriptional regulatory biology places numerous demands on optimization and inference machineries and limits scale. However, the field of deep learning offers scalable learning and optimization techniques that could aid in GRN inference. Deep learning has been used to model expression and covariance networks [16], to build a sparse representation of gene clusters [17], to group genes into co-regulated modules [18, 19], to do supervised clustering of gene sets [20, 21] and for dimensionality reduction and denoising[22]. However, interpretable deep learning models for gene regulation, which provide biological insight into causal relationships in addition to prediction, have been difficult to construct and is an active area of research [23]. Techniques to interpret deep-learning latent features often focus on removing latent features to quantify their influence and importance [24]. Feature quantification can use change in mean squared error on removal of input features (COM) [25], backpropagation of the output layer into latent features (Grad-CAM) [26], or forward propagation of the latent layers into the output layer [19]. These methods often require a target value e.g. as in classification tasks, or can’t be compared reliably between output features as non normalized error rates depend on feature scaling. These techniques cannot be used for model pruning or selection. To make statements on the active GRN in a given context we need to be able to determine predictive and non predictive interactions. For unbounded metrics this remains a problem. Deep learning models generally remain over-parameterized, making biological interpretation difficult, and techniques must be applied after training to eliminate model parameters and enforce sparsity for GRN evaluation[27–35].

Knowledge priming embeds existing structural evidence into the model architecture by limiting connectivity between features based on epigenetic or regulatory evidence found in the literature. This informs the model of constraints on regulation which are complementary and not directly measured by the readout of gene expression in the training data. These constraints allow direct interpretation of the model, as priming with biological interactions allows biological interpretation of the resulting network graph [36]. Other structure evidence embedding methods use this prior data type as a constraint on covariance [37]. Both of these methods lack the ability to infer novel regulatory structure.

With highlights on the most relevant parts.

I appreciate the benchmarking against GRN datasets and the detailed discussion of unique predictions. However, it would be interesting to see a more statistical analysis of TF activities, perhaps by examining correlations between TFA estimations in disease datasets and disease associations of the underlying TFs. This could further strengthen the method's applicability to genome medicine applications.

We thank the reviewer for this suggestion. To explore this idea we used the peripheral blood mononuclear cell (PBMC) model that was constructed in the original manuscript to interpret a COVID-19 single cell sequencing dataset containing healthy and diseased individuals.

We embedded it in the model, computing TFA for all cells. We then estimated differently active TFs based on their TFA estimate for each cell type as well as globally across the whole data. This analysis is summarized in supplemental figure 12 for the top 2 differently active TFs in each cell type for each condition comparing healthy and disease, and a select number of TFs is described in the result section 2.6. last paragraph.

It would be intriguing to explore the impact of documented TF interactions on the learning process. For example, using protein-protein interaction (PPI) data as a potential prior for estimating duplex activities.

This is a very interesting idea, and we agree with the reviewer. In fact, this was something that we considered (and we do believe that this is an avenue of research that will be productive in the future).

However, our initial exploration suggested that there is a great deal of work to be done to construct a reliable, genome-scale TF PPI prior, and that explicitly incorporating this prior into a deep-learning GRN model, so that the model can be interpretable for physical interactions, will not be trivial. We expect that this idea, by itself, would make a full standalone manuscript and we don’t think it is possible to properly address it as an add-on to the current manuscript.


Some minor comments:
a. Page 5, line 39: The notation should be corrected to L2 ∈ 10^−6 − 10^−4.

This is corrected to
L2 ∈ [10−6, 10−4]

b. Page 10, line 38 (Fig. 5B): Clarification is needed regarding mTF 227, as the gene ratio for associated genes related to the Phagosome pathway appears minimal (<0.01?), and the P-value is too small to discern.

We have now added a note in the figure text clarifying this data point. And it is corrected p value=0.0081, and gene ratio = 0.0309. To clarify we added this data to the figure legend in figure 5B.

The page numbers refer to the manuscript's page numbers.
Overall, this work represents a vital advancement in GRN inference, and with some additional clarification and analysis, it can further strengthen its impact on the field.


Reviewer #2:

In the manuscript authored by Tjarnberg et al, a novel GRN prediction method, SupirFactor, is proposed. SupirFactor uses target gene expressions and regulatory links and functional pathway information as prior information to learn gene regulatory networks (GRN) and TF activities (TFA) by autoencoder. The authors have performed various benchmarking experiments to evaluate the prediction accuracy of SupirFactor and functional interpretations of SupirFactor results. This method provides a new way of quantifying TF activities from expression data. However, the TFA itself is not a clearly defined concept (see below my comments). The importance of TFA therefore remains unclear to me with respect to what part of regulatory activity (transcription, splicing, translation, TF binding kinetics) it may help to give better interpretation than other methods. I would like to invite the authors to consider the following comments.

Major
One central question for TFA is how it is defined. It is fine to claim that TFA is difficult to measure experimentally. But it is important to define what it represents. Does it represent abundance of nascent mRNAs or any types of transcription/splicing/translation/binding kinetics for TFs? There should be some discussions or analyses to spell out its biological relevance.

The biological basis for Transcription Factor Activity can be summarized as “how much this transcription factor influences the transcriptional rate of Pol II at target genes”. This aggregates several properties - the TF gene transcription and processing into mature mRNA, the TF mRNA transcript translation into properly folded TF protein, the TF protein binding to its DNA target (e.g. localizing to the nucleus), inhibition in DNA binding or Pol II activation by a PTM or by a small molecule, etc. It’s possible to measure these TF properties in isolation, but we do not have the ability to measure TFA, as defined, from an entire organism for any specific TF (although if we could, the TFA unit would be a rate of nascent transcripts produced per minute per target gene).

We have improved the introduction of TFA in the second paragraph of the Background section to more clearly state which biophysical properties it aggregates, and the new section follows:

TFs themselves can be regulated transcriptionally (by changing their mRNA levels), translationally (by changing the amount of protein produced from mRNA), or post-translationally (by modifying the protein to alter localization or DNA binding ability).
Transcription factor activity (TFA) can be thought of as a representation of how much a transcription factor influences the transcriptional rate of Pol II at its target genes, theoretically measured as the marginal rate of nascent transcripts produced per minute per target gene. To have activity, the TF protein must be itself expressed, and it must be able to localize to the nucleus and bind to its DNA target. Depending on the TF, it may require specific post-translational modifications, presence or absence of a small molecule, and presence or absence of co-activators or co-repressors. This TF activity is difficult to measure experimentally genome-wide.


I think the biggest concern is that the ability to reconstruct target gene expressions does not necessarily indicate the true regulatory activities. The success of reconstruction may merely mean the model can learn how to remove the arbitrary noise from prior TF-target information, which may be the systematic bias within one dataset. In this case, holding-out part of the gold-standard GRN can give you good results but the inferred GRN might not be well generalizable. Other than hold-out validation using gold-standard GRNs, further experimental evidence(s) is needed. Such experimental evidence should be totally independent of the prior information used in generating the GRNs.

This is a valid concern. We think we’ve addressed dataset-specific biases, as we report the results of five entirely independent datasets in Figure 2, covering two species, bulk data (both RNA-seq and microarray), and single cell data.

Bias originating from the prior knowledge is also possible. It’s possible to try addressing this experimentally, but we are very reluctant to validate single network edges, as it’s difficult to know how well results from a handful of edges generalize (it’s also difficult to know as a reader if the edges chosen were chosen in an unbiased way - a good biologist would be able to choose edges that are most likely to validate from a candidate pool before doing any bench work). Unfortunately, our experimentalist colleagues tend to prefer limited-scale validation experiments, and that makes validating large chunks of a predicted organism-scale GRN impossible.

One of the major strengths of this work is the careful benchmarking on model organisms. We’ve used the regulatory network edges from these model organisms that we’re most confident in to make the gold standard and prior knowledge networks reported in the manuscript. There is a great deal of experimental evidence for other regulatory network edges which did not meet our rigorous threshold.

To create an additional test GRN, we looked at a second source of interactions, the YeastGenome regulation database and aggregated these edges and masked out any regulatory network edge which was present in existing prior knowledge or gold standard GRN. This new test GRN has absolutely zero edges which are provided to our model during training, but the edges still have some published experimental support. We think that this is the best metric for generalizability, as the model is entirely naive to this model.

We evaluate our model results on this test GRN and report the results in Figure 2F. We also compare to the alternative methods suggested in point 3. With a summary in the final paragraph of section 2.3 and a description of the data in the methods section 5.6.3. Summary reading:
Finally, we wanted to validate on regulatory edges not in the prior. We assembled an independent set of regulatory edges (section 5.6.3), and removed the overlap with the prior used for training. With this independent set of regulatory evidence we evaluated each method's model performance with AUPR. We found that the prior based method models outperforms the other methods tested for the independent set (Figure 2F).

Although the authors claimed that TF expressions do not truly reflect TF activities. I agree with this. However, one common (and simple) way to construct GRN is still to train a model to predict target expressions using TF expressions. The benchmark experiment may need to include at least one of these methods to show SupirFactor's advantage (e.g. GENIE3, GRNboost2).

This is an excellent suggestion. We have applied both GENIE3 and GRNBoost2 to the model organism benchmarking problems, and reported the results in Figure 2C and 2F.


The choice of informative genes as inputs is important for modeling. But the manuscript does not discuss how informative genes were selected. How were informative genes selected so as to reflect TFAs? This should be discussed.

We define informative genes as genes which are known to be regulated by a specific TF, and we call this the prior knowledge network when it is aggregated into a GRN.
We modified the text in the first paragraph of the results to read:

“This model ties the latent TF layer causally to informative genes, genes that have known upstream regulators given an independent set of evidence derived from literature or epigenetic data as detailed in section 2.1, and allows the activation of latent features in this layer to be directly interpreted as transcription factor activity (TFA).”

We also highlight what the informative genes mean when describing the prior matrix in section 2.2 with the addition;

“This data package also includes a B. subtilis gold standard network [10], covering 154 TFs and 4218 informative genes also used as the prior evidence, and a S. cerevisiae gold standard network [42], covering 98 TFs and 993 target genes, both derived from literature databases, we also use a S. cerevisiae prior evidence matrix with 150 TFs and 5578 informative genes used in previous work[41]”

Minor
1. Is the inferred GRN only a subset of the prior GRN? The training process will iteratively prune the links that does not have good xi^2 scores. Are these removed links all from original prior GRN or SupirFactor can prune links not present in the prior GRN? Can this method discover new links not in the prior GRN?

This model is not constrained to regulatory edges from the prior GRN. Every reported AUPR result in the manuscript is scored against a set of genes that were entirely held out of the prior GRN for validation. The iterative pruning is done on the inferred GRN that originally contain all interactions and in the manuscript for the models used for biological insight 1 pruning step is used with re-fitting.

2. In 2.3, the authors stated that max R2 improves the R2 at a cost of decreasing AUPR, but max R2 actually has higher AUPRs in fig 2A and fig 2B.

We observe that the behavior is slightly different between the bulk datasets performance and the single cell dataset when using ERV. For Figure 2A (bulk data) we maintain that this observation holds Which is the point we emphasize in the first paragraph. But for ERV in single cell, Figure 2B, we find this relationship to be dependent on other factors like preprocessing, as well as having a higher variability in general. We guide our hyper-parameter settings from insight of the general trends of our benchmark.

3. The authors showed that RobustMinScaler outperformed StandardScaller and sparsity of RobustMinScaler gave the advantage. This is interesting. Can this be caused by the fact that standardization is unbound? The zeros of each gene got transformed into different negative values then this can confuse the model, introducing technical noise.

This is an interesting question and this is still something we are exploring. We thought about a potential reason for this. We agree with the reviewer that this is probably related to how signal is propagated in the model and how activation functions are influencing latent activity. The model needs to compensate for a non-zero baseline for StandardScalar data.

4. Why testing R^2 is higher than training R^2 in fig. 2E?

The training dataset contains fifteen individual single-cell experiments from two different published datasets. The test R2 data is reported for two additional experiments, which have less total variance than the combined training data.

5. In section 2.5 the authors stated that " and we see that these conditions have very similar mTF activation (Figure 4B)". The authors should specify the exact groups they referred to.

We have clarified this in the text as follows:and we see that these conditions have similar mTF activation (for mTFs; 78, 72, 57, 88, 64, 116) within the glucose conditions (Figure 4B)

6. In 4.3.1, the penalty strength is set to be identical to W, theta and PI. What if the three parts are penalized differently?

This is a very interesting question. In this work we opted to not explore this extra dimension of hyper parameters as the combinatorial complexity grew rapidly for the number of possible hyper-parameters in the deep learning frameworks. We expect that future work will explore additional model structures.

7. More details should be given to mTF layer. Does mTF layer have the same number of neurons as TF layer? Also details about number of neurons in each layer should be specified.

We have now clarified the layer widths in section 2.2 first paragraph and section 2.4 first paragraph.

**Second round of review**

**Reviewer 1**

The authors have addressed my questions and I am happy to see it getting published.

**Reviewer 2**

In this version of revision, the author has substantially revised the analyses and the manuscript. My comments have been properly addressed. The manuscript could be accepted in its current form.
